# Geographic patterns of genomic diversity and structure in the C_4_ grass *Panicum hallii* across its natural distribution

**DOI:** 10.1093/aobpla/plab002

**Published:** 2021-01-06

**Authors:** Juan Diego Palacio-Mejía, Paul P Grabowski, Edgardo M Ortiz, Gustavo Adolfo Silva-Arias, Taslima Haque, David L Des Marais, Jason Bonnette, David B Lowry, Thomas E Juenger

**Affiliations:** 1 Department of Integrative Biology, The University of Texas at Austin, Austin, TX, USA; 2 Genome Sequencing Center, HudsonAlpha Institute for Biotechnology, Huntsville, AL, USA; 3 Ecology & Ecosystem Management, Plant Biodiversity Research, Technical University of Munich, Freising, Germany; 4 Professorship for Population Genetics, Department of Life Science Systems, School of Life Sciences, Technical University of Munich, Freising, Germany; 5 Civil and Environmental Engineering, Massachusetts Institute of Technology, Cambridge, MA, USA; 6 Department of Plant Biology, Michigan State University, East Lansing, MI, USA

**Keywords:** ddRAD-seq, ecological genomics, evolution, genetic admixture, habitat suitability modelling, *Panicum*, phylogeographic structure

## Abstract

Geographic patterns of within-species genomic diversity are shaped by evolutionary processes, life history and historical and contemporary factors. New genomic approaches can be used to infer the influence of such factors on the current distribution of infraspecific lineages. In this study, we evaluated the genomic and morphological diversity as well as the genetic structure of the C_4_ grass *Panicum hallii* across its complex natural distribution in North America. We sampled extensively across the natural range of *P. hallii* in Mexico and the USA to generate double-digestion restriction-associated DNA (ddRAD) sequence data for 423 individuals from 118 localities. We used these individuals to study the divergence between the two varieties of *P. hallii*, *P. hallii* var. *filipes* and *P. hallii* var. *hallii* as well as the genetic diversity and structure within these groups. We also examined the possibility of admixture in the geographically sympatric zone shared by both varieties, and assessed distribution shifts related with past climatic fluctuations. There is strong genetic and morphological divergence between the varieties and consistent genetic structure defining seven genetic clusters that follow major ecoregions across the range. South Texas constitutes a hotspot of genetic diversity with the co-occurrence of all genetic clusters and admixture between the two varieties. It is likely a recolonization and convergence point of populations that previously diverged in isolation during fragmentation events following glaciation periods.

## Teaser

Geographic patterns of diversity are shaped by historical and contemporary factors that drive evolutionary processes. Genomic approaches can be used to infer the influence of such factors on the current distribution of genetic variation. We studied diversity of the C_4_ grass *Panicum hallii* across its native range in North America. We observed strong genetic and morphological divergence across seven genetic clusters that follow major ecoregions. South Texas is a hotspot of genetic diversity with the co-occurrence of all genetic clusters and admixture between two described varieties. South Texas is likely a recolonization and convergence point of formerly diverged populations.

## Introduction

Genetic variation is the raw material necessary to understand evolution and adaptation to diverse environmental conditions (Conner and Hartl 2004; Randall *et al.* 2008; Jump *et al.* 2009). Evolutionary processes generate new diversity through random mutations which are under the influence of microevolutionary forces such as genetic drift, migration and selection (Vellend and Geber 2005). A plant species’ life history, including breeding system and dispersal mechanisms, has an influence on the genetic diversity and distribution of species (Hamrick and Godt 1996; Ellegren and Galtier 2016). Current plant species distributions are the result of geological events that have had influence on soil patterns and changes in climate. For example, the soil composition of the Atlantic coastal plains in North America has been influenced by sediment deposits from the Late Cretaceous to recent deposits from the Pleistocene (Noss *et al.* 2015). Also, climatic fluctuations during the Quaternary have caused the contraction and expansion of species distribution, with remarkable genetic consequences (Hewitt 2000). Shifts in range distributions can result in the loss of alleles because of bottlenecks during range expansion or the introgression of novel allele by gene flow from secondary contacts of genetic lineages differentiated in allopatry during the contractions (Hewitt 2001, 2004). Finally, the current ecological conditions covering a species’ distribution can affect genetic structure as a result of local adaptation to these environments. Linking these processes across spatial and temporal scales can help identify the drivers of the current genetic structure of plant populations.

**Figure 1.**
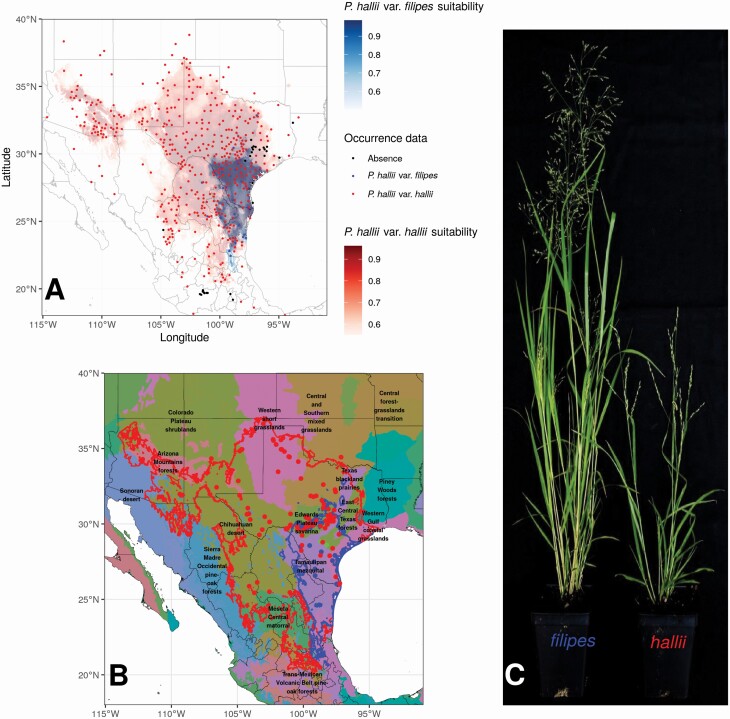
Natural distribution of *Panicum hallii*. (A) Map of *P. hallii* distribution inferred from ensemble HSM and occurrence points from personal field collections and observations and secondary records from the Global Biodiversity Information Facility (GBIF.org (24 April 2014) GBIF Occurrence Download https://doi.org/10.15468/dl.bqb4uz) and herbarium visits. The map also shows confirmed absence locations of *P. hallii*. Detailed maps of the projections of the ensemble habitat suitability models can be found in the **Supporting Information—Figs S8**  **and**  **S12**. (B) Map of collections of *P. hallii* used for genetic and morphological analyses. The map includes the ecoregions showing the ecological preferences for *P. hallii* and the shaded area of the habitat suitability models. The East boundary of the distribution of *P. hallii* matches with the East Central Texas forest, where several failed collection trips were made. The South distribution was limited to the Meseta Central Matorral in Mexico (Olson *et al.* 2001). Red dots represent var. *hallii*, blue dots var. *filipes*, green dots localities where both varieties were collected and black dots correspond to true absences. (C) *Panicum hallii* varieties grew under greenhouse conditions, to the left variety *filipes* and to the right variety *hallii*.

Here, we explored the genetic effect of different environmental factors on the genetic variation of the Hall’s panicgrass, *Panicum hallii* at the population level. This species is a perennial C_4_ grass native to North America with a distribution that spans from the south-eastern part of Mexico through the South-Central and South-western regions of the USA (Fig. 1A). In its natural range, *P. hallii* is found across several environmental gradients such as mesic to xeric (east to west), semi-tropical to temperate hardiness zones (south to north) and altitudinal (from sea level along the coastal shore of the Gulf of Mexico to over 2200 metres above sea level in the Guadalupe Mountains, Texas). This species is also found in a variety of soil and ecological conditions, including nine ecoregions as defined by Olson *et al.* (2001) (Fig. 1B). The species has two varieties that are morphologically well-differentiated: the widespread *P. hallii* var. *hallii* (hereafter var. *hallii*) and the more restricted *P. hallii* var. *filipes* (hereafter var. *filipes*). Both varieties occur sympatrically in a small part of their ranges. Variety *hallii* has a native distribution that extends from southern Colorado south into Mexico and from western Arizona to eastern Texas, and typically occurs on a variety of soil substrates, including sandy to shallow, dry, rocky and calcareous soils. In contrast, variety *filipes* is typically found in areas of transition between clay soils and mesic depressions along the Western Gulf Coastal Grassland ecoregion and the Pine Oak Forest ecoregion at the Rio Grande Valley (Waller 1976; Lowry *et al.* 2013). In these two ecoregions of south-central Texas, both varieties can be found in sympatry. Although *P. hallii* is a highly inbred species (Lowry *et al.* 2012), hybrids of both varieties can be obtained under controlled conditions in the greenhouse (Lowry *et al.* 2015). Nevertheless, the existence of hybridization between varieties in natural populations is unknown.

The two varieties of *P. halli* were described at the end of the 19th century as two different species, *P. hallii* and *P. filipes*. A century later, in a taxonomic treatment of the species of *Panicum* section *Diffusa*, the taxonomic status of these two species changed to varieties of *P. hallii* based on panicle morphology (Waller 1974) with clear morphological separation between the varieties. The *filipes* variety is generally larger than var. *hallii* with the exception of seed size (Waller 1976). More recently, a set of 18 specific microsatellite markers was developed and validated for *P. hallii* (Lowry *et al.* 2012), revealing a low heterozygosity in the species, which suggests a self-fertilization pollination system (Lowry *et al.* 2013). Morphological and genetic analyses of 39 populations collected along a longitudinal transect from the deserts of Arizona and New Mexico through the savannas of Central Texas showed strong geographic population structure for var. *hallii* (mean *F*_ST_ = 0.6) with a split that occurs in western Texas (Lowry *et al.* 2013). Beyond these two studies, little is known about the drivers of genomic diversity of *P. hallii* across its native geographical range.

Here we combine genomics, environmental niche models and common garden experiments using more than 400 samples to characterize the patterns and distribution of diversity in *P. hallii* across its natural range. This includes 351 newly collected samples, incorporating novel material from Mexico, West and Central Texas, and natural collections from four close relative species. We used both double-digestion restriction-associated DNA (ddRAD), a reduced representative genotyping method, and seven discriminant morphological markers to address three major questions: (i) What is the degree of divergence between the two varieties of *P. hallii*? (ii) How is the genomic and morphological diversity distributed within each variety? (iii) Is there evidence of admixture between individuals from these two varieties, especially when they occur in sympatry? Answering these evolutionary questions will help us to understand how evolutionary and ecological processes have shaped species diversity.

## Materials and Methods

### Plant material collection

The plant material used in this study comes from two sources. First, we used *P. hallii* seed collections derived from previous studies (Lowry *et al.* 2012, 2013), which provides a representative sampling of the species distribution from the South-western USA. Second, we conducted new field collections between 2012 and 2015 in Mexico and several Texas ecoregions (Western Gulf Coastal Grasslands, Pine Oak Forest, East Central Texas forest, Texas Blackland Prairies, Chihuahuan Desert and Edwards Plateau Savanna), adding 351 new collections to the 298 previously collected plants. These new collections complement the previous collection effort and provide an exhaustive sampling across the natural distribution of this species.

Newly collected seeds and whole plants were transported to The University of Texas at Austin greenhouse facilities for propagation. Herbarium vouchers were collected and deposited in the Billie L. Turner Plant Resources Center at The University of Texas at Austin, and leaf tissue was collected from these plants and stored in a −80 °C freezer for DNA extraction. Seeds from the collected plants were harvested and stored in a seed collection. Overall, we include 649 samples from these collections in this study (Table 1; Fig. 1B; **see**  **Supporting Information—Fig. S1**).

**Table 1.** List of localities. *N* = number of samples; *H*_e_ = expected heterozygosity.

Loc ID	Species	Region	Country	State	County	Altitude m a.s.l.	Latitude	Longitude	*N*	*H*_e_
HAR	*Panicum capillare*		USA	Texas	Harris	6	29.7197	−94.9502	5	
DSO	*Panicum diffusum*		USA	Louisiana	De Soto	51	32.3062	−93.8076	1	
ENR	*Panicum diffusum*		USA	Texas	Llano	461.3	30.5085	−98.8246	4	
ARA	*Panicum hallii* var. *filipes*	Coastal and inland	USA	Texas	Aransas	2	28.2237	−96.987	1	0.044
AWR	*Panicum hallii* var. *filipes*	Coastal	USA	Texas	Aransas	2.7	28.2851	−96.9457	2	0.076
CAM	*Panicum hallii* var. *filipes*	Coastal and inland	USA	Texas	Cameron	5	26.0007	−97.2691	3	0.039
DIM	*Panicum hallii* var. *filipes*	Inland	USA	Texas	Dimmit	162	28.5701	−99.5245	1	0.025
EPS	*Panicum hallii* var. *filipes*	Coastal and inland	USA	Texas	Maverick	248	28.4826	−100.21	1	0.041
FIL	*Panicum hallii* var. *filipes*	Coastal and inland	USA	Texas	Nueces	3	27.6496	−97.4039	7	0.059
LAR	*Panicum hallii* var. *filipes*	Coastal and inland	USA	Texas	Webb	160	27.4773	−99.2092	3	0.063
NDP	*Panicum hallii* var. *filipes*	Coastal	USA	Texas	San Patricio	1	27.9117	−97.6076	3	0.052
PAB	*Panicum hallii* var. *filipes*	Coastal	USA	Texas	Cameron	9	26.0199	−97.4745	1	0.054
ZAF	*Panicum hallii* var. *filipes*	Inland	USA	Texas	Zapata	97	26.5759	−99.1091	1	0.04
ZAP	*Panicum hallii* var. *filipes*	Coastal	USA	Texas	Zapata	116	27.021	−99.1251	3	0.043
GNF	*Panicum hallii* var. *filipes*	Coastal	USA	Texas	Dewitt	76.5	29.3119	−97.3001	2	0.038
MCM	*Panicum hallii* var. *filipes*	Coastal and inland	USA	Texas	McMullen	100	28.1519	−98.6385	1	
NUE	*Panicum hallii* var. *filipes*	Coastal	USA	Texas	Nueces	15	27.5684	−97.7956	1	
ATC	*Panicum hallii* var. *filipes*	Inland	USA	Texas	Atascosa	786	28.824	−98.6932	5	0.02
ATC	*Panicum hallii* var. *hallii*	Sympatric	USA	Texas	Atascosa	786	28.824	−98.6932	5	0.016
RAR	*Panicum hallii* var. *hallii*	Sympatric	USA	Texas	Live Oak	83	28.607	−98.222	5	0.034
RAR	*Panicum hallii* var. *filipes*	Inland	USA	Texas	Live Oak	83	28.607	−98.222	5	0.024
ABI	*Panicum hallii* var. *hallii*	Central Texas	USA	Texas	Coleman	540	31.8599	−99.4422	3	0.006
ALI	*Panicum hallii* var. *hallii*	West	USA	Texas	Potter	961	35.5739	−101.7011	7	0.004
ARB	*Panicum hallii* var. *hallii*	Central Texas	USA	Oklahoma	Murray	305	34.4298	−97.1467	4	0.007
ART	*Panicum hallii* var. *hallii*	West	USA	New Mexico	Chaves	1441	32.8567	−104.9316	10	0.004
ATA	*Panicum hallii* var. *hallii*	Tex-Mex	USA	Texas	Atascosa	113	28.7897	−98.5413	1	0.015
ATO	*Panicum hallii* var. *hallii*	Tex-Mex	Mexico	Durango	Mezquital	1869	23.693	−104.3961	5	0.027
BAN	*Panicum hallii* var. *hallii*	Central Texas	USA	Texas	Bandera	679	29.8473	−99.5734	5	0.008
BBD	*Panicum hallii* var. *hallii*	Tex-Mex	USA	Texas	Brewster	780	29.6235	−103.1162	4	0.014
BBF	*Panicum hallii* var. *hallii*	Tex-Mex	USA	Texas	Brewster	1244.4	29.2736	−103.3375	3	0.011
BEE	*Panicum hallii* var. *hallii*	Sympatric	USA	Texas	Bee	83	28.3828	−97.7792	4	0.034
BEL	*Panicum hallii* var. *hallii*	Central Texas	USA	Texas	Bell	240	30.9183	−97.6581	5	0.006
BEX	*Panicum hallii* var. *hallii*	Central Texas	USA	Texas	Bexar	341	29.7214	−98.3542	5	0.005
BME	*Panicum hallii* var. *hallii*	West	USA	Oklahoma	Cimarron	1319	36.8753	−102.8874	6	0.005
BOS	*Panicum hallii* var. *hallii*	West	USA	Texas	Bosque	204	32.128	−97.5596	5	0.006
BSP	*Panicum hallii* var. *hallii*	Sympatric	USA	Texas	Brown	440	31.8616	−99.0258	1	0.015
BUR	*Panicum hallii* var. *hallii*	Sympatric	USA	Texas	Burnet	248	30.5468	−98.1562	5	0.013
BUS	*Panicum hallii* var. *hallii*	West	USA	Texas	Potter	1119	35.2649	−102.0605	8	0.004
CBS	*Panicum hallii* var. *hallii*	Central Texas	USA	Texas	San Saba	346.4	31.0574	−98.4839	5	0.008
CCG	*Panicum hallii* var. *hallii*	West	USA	Texas	Briscoe	754	34.4468	−101.0823	6	0.006
CCL	*Panicum hallii* var. *hallii*	West	USA	Texas	Briscoe	742	34.4441	−101.0751	5	0.006
CCO	*Panicum hallii* var. *hallii*	West	USA	Texas	Briscoe	749	34.4379	−101.0561	2	0.005
CDM	*Panicum hallii* var. *hallii*	Tex-Mex	Mexico	Coahuila	Parras	1262	25.5083	−102.5699	5	0.016
CHA	*Panicum hallii* var. *hallii*	West	USA	Texas	Oldham	1069	35.6052	−102.2966	8	0.004
CKR	*Panicum hallii* var. *hallii*	West	USA	Arizona	Maricopa	1817	34.2342	−112.3154	10	0.004
CLA	*Panicum hallii* var. *hallii*	West	USA	New Mexico	Union	1463	36.6364	−103.0375	1	0.004
CLD	*Panicum hallii* var. *hallii*	West	USA	Texas	Armstrong	956	34.7984	−101.4363	7	0.004
CNF	*Panicum hallii* var. *hallii*	West	USA	Arizona	Santa Cruz	1550	31.8021	−110.9152	4	0.004
COL	*Panicum hallii* var. *hallii*	Sympatric	USA	Texas	Coleman	474	31.8063	−99.2348	5	0.013
COM	*Panicum hallii* var. *hallii*	Tex-Mex	USA	Texas	Val Verde	485	29.7018	−101.2077	2	0.013
COR	*Panicum hallii* var. *hallii*	Tex-Mex	USA	Arizona	Maricopa	1110	34.2862	−112.179	17	0.01
CRC	*Panicum hallii* var. *hallii*	West	USA	Texas	Oldham	985	35.5298	−102.2651	8	0.005
CRS	*Panicum hallii* var. *hallii*	West	USA	Texas	Briscoe	740.6	34.4374	−101.0568	4	0.005
DOF	*Panicum hallii* var. *hallii*	Tex-Mex	USA	Texas	Val Verde	417	29.8946	−100.9946	7	0.017
DRB	*Panicum hallii* var. *hallii*	Tex-Mex	USA	Texas	Val Verde	477	29.9412	−100.9706	5	0.015
DRS	*Panicum hallii* var. *hallii*	Tex-Mex	USA	Texas	Val Verde	457.4	29.9051	−101.0032	5	0.023
DUV	*Panicum hallii* var. *hallii*	Sympatric	USA	Texas	Duval	139	27.9141	−98.7002	3	0.012
EDW	*Panicum hallii* var. *hallii*	Central Texas	USA	Texas	Edwards	628	29.8263	−100.4664	5	0.006
ELG	*Panicum hallii* var. *hallii*	West	USA	Arizona	Santa Cruz	1531	31.6009	−110.5585	10	0.005
ESE	*Panicum hallii* var. *hallii*	Central Texas	USA	Texas	Kimble	642	30.3482	−99.5894	3	0.01
GEO	*Panicum hallii* var. *hallii*	West	USA	New Mexico	Harding	1780	36.0429	−104.3092	1	0.006
GIL	*Panicum hallii* var. *hallii*	West	USA	New Mexico	Grant	2101	32.8273	−108.057	25	0.003
GMO	*Panicum hallii* var. *hallii*	West	USA	Texas	Culberson	2257.5	31.9018	−104.8391	6	0.006
GRA	*Panicum hallii* var. *hallii*	West	USA	Texas	Kerr	642	29.9149	−99.2431	1	0.016
HAL	*Panicum hallii* var. *hallii*	Central Texas	USA	Texas	Travis	244	30.185	−97.874	2	0.007
HAY	*Panicum hallii* var. *hallii*	Central Texas	USA	Texas	Hays	387	29.9653	−98.1804	6	0.004
HWY	*Panicum hallii* var. *hallii*	West	USA	Texas	Briscoe	931	34.4691	−101.1103	5	0.004
JHC	*Panicum hallii* var. *hallii*	Tex-Mex	USA	Texas	Blanco	361	30.2073	−98.307	4	0.02
JWE	*Panicum hallii* var. *hallii*	Sympatric	USA	Texas	Jim Wells	169	27.894	−98.0055	1	0.014
KEN	*Panicum hallii* var. *hallii*	Tex-Mex	USA	Texas	Kendall	478	29.8593	−98.694	5	0.009
KER	*Panicum hallii* var. *hallii*	Central Texas	USA	Texas	Kerr	806	30.0397	−99.3973	5	0.009
KNT	*Panicum hallii* var. *hallii*	West	USA	Texas	Culberson	1326	31.0499	−104.212	3	0.006
LBY	*Panicum hallii* var. *hallii*	West	USA	New Mexico	Guadalupe	1450	34.945	−104.6406	3	0.005
LMA	*Panicum hallii* var. *hallii*	Central Texas	USA	Texas	Real	678	30.0164	−99.7314	4	0.007
LME	*Panicum hallii* var. *hallii*	West	USA	Texas	Hutchinson	918	35.7073	−101.5465	7	0.006
LOS	*Panicum hallii* var. *hallii*	West	Mexico	Coahuila		1550	25.4619	−101.0239	3	0.01
MAP	*Panicum hallii* var. *hallii*	Tex-Mex	Mexico	Durango	Mapimi	1563	25.872	−104.1051	5	0.014
MCR	*Panicum hallii* var. *hallii*	West	USA	Texas	Jeff Davis	1865	30.6829	−104.1348	8	0.007
MEZ	*Panicum hallii* var. *hallii*	Tex-Mex	Mexico	Durango	Mezquital	1600	23.4945	−104.4324	5	0.01
MKF	*Panicum hallii* var. *hallii*	Central Texas	USA	Texas	Travis	174	30.1777	−97.7251	2	0.044
NDD	*Panicum hallii* var. *hallii*	Tex-Mex	Mexico	Durango	Nombre de Dios	1866	23.8979	−104.335	5	0.013
NMX	*Panicum hallii* var. *hallii*	West	USA	New Mexico	Dona Ana	1656	32.4299	−106.5815	2	0.005
NOH	*Panicum hallii* var. *hallii*		USA	Texas	Willacy	4.57	26.4949	−97.5453	1	0.021
PAJ	*Panicum hallii* var. *hallii*	Sympatric	USA	Texas	Kimble	532	30.4854	−99.7376	1	0.019
PCR	*Panicum hallii* var. *hallii*	West	USA	Arizona	Cochise	1652	31.9802	−109.3634	1	0.004
PDB	*Panicum hallii* var. *hallii*	West	USA	Texas	Randall	870	34.9287	−101.6366	9	0.004
PDF	*Panicum hallii* var. *hallii*	West	USA	Texas	Randall	864	34.9437	−101.6613	8	0.003
PDK	*Panicum hallii* var. *hallii*	West	USA	Texas	Randall	905	34.9476	−101.689	1	0.004
PDL	*Panicum hallii* var. *hallii*	West	USA	Texas	Randall	892	34.9476	−101.689	2	0.003
PDS	*Panicum hallii* var. *hallii*	West	USA	Texas	Randall	860	34.9395	−101.6528	2	0.004
PDT	*Panicum hallii* var. *hallii*	West	USA	Texas	Randall	1040	34.9391	−101.6305	8	0.004
PFL	*Panicum hallii* var. *hallii*	Central Texas	USA	Texas	Travis	200	30.4506	−97.6386	2	0.006
PIN	*Panicum hallii* var. *hallii*	West	USA	Arizona	Gila	1642	34.3161	−111.4006	3	0.003
PIS	*Panicum hallii* var. *hallii*	Sympatric	USA	Texas	Cameron	5	26.0173	−97.2735	1	0.009
POI	*Panicum hallii* var. *hallii*	Sympatric	USA	Texas	Cameron	5	26.0643	−97.2375	2	0.012
PRT	*Panicum hallii* var. *hallii*	West	USA	Arizona	Cochise	1478	31.9159	−109.1547	8	0.003
REA	*Panicum hallii* var. *hallii*	Central Texas	USA	Texas	Real	599	29.7683	−99.8171	5	0.008
SAN	*Panicum hallii* var. *hallii*	Tex-Mex	USA	Texas	Terrel	981	30.1346	−102.5633	3	0.007
SAP	*Panicum hallii* var. *hallii*	Tex-Mex	Mexico	Durango	Lerdo	1476	25.4331	−103.7161	5	0.016
SCG	*Panicum hallii* var. *hallii*	Tex-Mex	USA	Texas	Val Verde	421.4	29.6942	−101.3221	5	0.014
SEM	*Panicum hallii* var. *hallii*	Tex-Mex	USA	Texas	Val Verde	425	29.681	−101.3099	4	0.016
SEP	*Panicum hallii* var. *hallii*	Central Texas	USA	Texas	Travis	291.9	30.4013	−97.797	7	0.018
SEV	*Panicum hallii* var. *hallii*	West	USA	New Mexico	Socorro	1544	34.3317	−106.9735	24	0.003
SLR	*Panicum hallii* var. *hallii*	Sympatric	USA	Texas	Kimble	538	30.4453	−99.7974	1	0.01
SLS	*Panicum hallii* var. *hallii*	Tex-Mex	USA	Texas	Burnet	391	30.9913	−98.1008	4	0.012
SMF	*Panicum hallii* var. *hallii*	Central Texas	USA	Texas	Lampasas	476	31.3052	−98.4387	1	0.007
SMT	*Panicum hallii* var. *hallii*	Tex-Mex	USA	Texas	Mills	488	31.5616	−98.5992	3	0.016
SNO	*Panicum hallii* var. *hallii*	West	USA	Arizona	Pima	1567	31.8005	−110.7035	8	0.004
SOM	*Panicum hallii* var. *hallii*	Central Texas	USA	Texas	Somervell	191	32.2756	−97.6016	5	0.007
SPE	*Panicum hallii* var. *hallii*	West	USA	Texas	Crockett	744	30.6664	−100.9707	23	0.005
SPJ	*Panicum hallii* var. *hallii*	Central Texas	USA	Texas	Kimble	602	30.47	−99.7308	5	0.006
SPR	*Panicum hallii* var. *hallii*	West	USA	Texas	Jeff Davis	1900	30.6723	−104.1277	1	0.006
SRG	*Panicum hallii* var. *hallii*	Tex-Mex	USA	Texas	Val Verde	380.5	29.6608	−101.3152	8	0.018
STW	*Panicum hallii* var. *hallii*	Central Texas	USA	Texas	Palo Pinto	332	32.6093	−98.4896	8	0.006
SVN	*Panicum hallii* var. *hallii*	West	USA	New Mexico	Guadalupe	1551	34.8311	−104.8274	9	0.004
THC	*Panicum hallii* var. *hallii*	Central Texas	USA	Texas	Travis	289	30.2419	−98.0091	5	0.014
TLA	*Panicum hallii* var. *hallii*	Tex-Mex	Mexico	Durango	Tlahualilo	1114	26.1281	−103.4992	5	0.012
TNK	*Panicum hallii* var. *hallii*	West	USA	New Mexico	Guadalupe	1398	34.9369	−104.694	4	0.004
TWC	*Panicum hallii* var. *hallii*	Central Texas	USA	Texas	Travis/Williamson	360	30.6222	−97.9719	5	0.004
UVA	*Panicum hallii* var. *hallii*	Sympatric	USA	Texas	Uvalde	304	29.2415	−100.0972	1	0.014
UVL	*Panicum hallii* var. *hallii*	Sympatric	USA	Texas	Uvalde	550	29.5983	−100.0253	2	0.037
WBW	*Panicum hallii* var. *hallii*	Sympatric	USA	Texas	Kimble	597	30.4329	−99.7958	3	0.045
ARR	*Panicum hallii* var. *hallii*	West	USA	Arizona	Santa Cruz	1460	31.6134	−110.5063	2	
CMR	*Panicum hallii* var. *hallii*	Sympatric	USA	Texas	Maverick	219	28.5835	−100.1117	2	
GNH	*Panicum hallii* var. *hallii*	Sympatric	USA	Texas	Dewitt	76.5	29.3119	−97.3001	2	0.029
HAM	*Panicum hallii* var. *hallii*	Tex-Mex	USA	Texas	Stonewall	505	33.0098	−100.1813	2	
HUE	*Panicum hallii* var. *hallii*	Sympatric	USA	Texas	Gillespie	550	30.2255	−99.0156	1	
KIC	*Panicum hallii* var. *hallii*	Tex-Mex	USA	Texas	Kinney	561	29.6185	−100.443	1	
MAV	*Panicum hallii* var. *hallii*	Sympatric	USA	Texas	Maverick	215	28.2386	−100.1592	3	
RAN	*Panicum hallii* var. *hallii*	West	USA	Texas	Lubbock	990	33.4602	−101.9143	2	
CHR	*Panicum lepidulum*		Mexico	Michoacan	Charo	1975	19.6908	−101.0312	5	
CUT	*Panicum lepidulum*		Mexico	Michoacan	Morelia	2113	19.7213	−101.3424	2	
ERO	*Panicum lepidulum*		Mexico	Michoacan	Erongaricuaro	2101	19.6072	−101.6871	5	
HTE	*Panicum lepidulum*		Mexico	Michoacan	Huaniqueo	2160	19.8959	−101.4332	3	
HUA	*Panicum lepidulum*		Mexico	Michoacan	Huaniqueo	2141	19.876	−101.4334	5	
MOR	*Panicum lepidulum*		Mexico	Michoacan	Morelia	1975	19.6761	−101.3136	5	
PTZ	*Panicum lepidulum*		Mexico	Michoacan	Patzcuaro	2128	19.5581	−101.5781	1	
TEM	*Panicum lepidulum*		Mexico	Estado de Mexico	Temamatla	2344	19.1887	−98.8921	5	
TEN	*Panicum lepidulum*		Mexico	Michoacan	Huaniqueo	2207	19.8947	−101.4284	3	
TZI	*Panicum lepidulum*		Mexico	Michoacan	Morelia	2127	19.7908	−101.4445	1	
ALD	*Panicum* sp.		Mexico	Tamaulipas	Aldana	269	22.863	−98.2296	1	

### Habitat suitability modelling

We implemented an ensemble modelling approach using the biomod2 package in R (Araújo and New 2007; Thuiller *et al.* 2009) to explore habitat suitability based on occurrence and climate data. Our approach included the model obtained with five cross-validation replicates of seven presence–absence algorithms and five pseudo-absence sampling (a total of 175 models). We implemented independent habitat suitability modelling (HSM) runs for var. *hallii* and var. *filipes* using presence records, *in situ* confirmed (real) absence locations, as well as pseudo-absence points sampled in the whole species distribution area **[see**  **Supporting Information—Fig. S2****]**. Considering the wide geographic distribution of var. *hallii* covering extensive environmental heterogeneity, we also performed additional HSMs for the two largest infraspecific genetic clusters (West and Tex-Mex; see Results). As explanatory variables, we included eight (largely independent) bioclimatic variables from the WorldClim v.1.4 (Hijmans *et al.* 2005). To evaluate the possible influence of historical and climate change processes on the observed patterns of genetic diversity and differentiation in *P. hallii*, we projected the HSMs onto three past climate scenarios including the mid-Holocene (~6 Kya), Last Glacial Maximum (LGM, ~22 Kya) and Last Inter-Glacial (LIG, 120–140 Kya) available in the WorldClim v.1.4 climatic database. For our distribution hindcast distribution analyses, we assessed the intrinsic uncertainty of the simulation of past climatic scenarios by projecting the HSMs using climatic data derived from three different global circulation models (GCMs). A complete description of the data and modelling methods can be found in the **Supporting Information 2**.

### Genotyping

High-quality DNA was extracted from the leaf tissue of each individual plant using a modified CTAB protocol (Allen *et al.* 2006). DNA concentration was quantified using a Qubit® 2.0 fluorometer and dsDNA BR Assay Kit (Life Technologies, Carlsbad, CA, USA). The quality and purity of the genomic DNA was evaluated by running the samples on a 1 % agarose gel for comparison to a low molecular weight ladder (New England BioLabs). All samples were normalized at 1 μg of DNA and stored at −20 °C until used.

In order to obtain a genome-wide representation of genetic diversity at low cost, we used ddRAD sequencing (Peterson *et al.* 2012). Based on *in silico* digestions (Lepais and Weir 2014; Mora-Márquez *et al.* 2017) of the *P. hallii* var. *filipes* reference genome v. 2.0 (DOE-JGI, https://phytozome.jgi.doe.gov), we chose the combination of NspI and Mlucl restriction enzymes for the ddRAD method.

DNA samples were submitted in three sets to the Genomics and Bioinformatics Service at Texas A&M University (College Station, TX, USA). Library preparation followed an in-house protocol. Briefly, for each sample, around 1 μg of genomic DNA was digested with the restriction enzymes NspI and Mlucl and then adaptor ligation fragments were selected in a range of 375–650 bp using Pippin Prep technology (Sage, Beverly, MA, USA). Finally, the library samples were sequenced using the Illumina HiSeq 2500 and 4000 (San Diego, CA, USA), producing around 2.6 billion of 2 × 100 or 2 × 125 pair-end raw reads.

### Filtering sequencing data for nucleotide polymorphism calling and quality control

We obtained a total of ~2.6 billion raw reads for the 649 samples submitted, with a mean of ~4 million pair-end raw reads per sample (mean = 3.961.472 ± 3.265.748). Raw fastq read quality was evaluated with *FastQC* v. 0.11.3 (Andrews 2018). Sequencing reads were pre-processed using BBTools v. 37.50 in five steps: (i) Trimming of adaptor sequence. (ii) Removal of reads corresponding to the phiX viral genome and other common contaminants. (iii) Trimming of the cutsite ‘AATT’. (iv) Merging of overlapping paired reads. (v) Trimming of low-quality bases, retaining reads of at least 35 bp after processing.

The filtered reads were mapped to the *P. hallii* var. *hallii* v. 2.0 reference genome (DOE-JGI, https://phytozome.jgi.doe.gov), using bbmap v. 37.50 (Bushnell 2014). We used the maximum likelihood statistical model in Stacks v. 1.47 (Catchen *et al.* 2011, 2013) to call SNPs, following the pipeline designed for ecology and population genomics (Rochette and Catchen 2017). The *populations* program in Stacks was used to divide the samples into three biological groups: one set for all *P. hallii* samples, one set for var. *hallii* and one set for var. *filipes*. We used a minimum minor allele frequency of 0.05 to process a nucleotide at a locus. We were concerned about poor mapping of short RAD reads given the repetitive aspects of many plant genomes. As such, we filtered for paralog loci by removing markers with an excess of heterozygosity and excluding heterozygous loci with strong allele ratio deviations using the *HDplot* method (McKinney *et al.* 2017). We removed markers with heterozygosity >6 %, which was the maximum heterozygosity obtained using microsatellite markers in this species (Lowry *et al.* 2013) and the read ratio deviation (1:4, *D* > 4) to minimize the presence of loci that consist of repetitive regions that have been co-assembled (da Fonseca *et al.* 2016). We used paralog-finder v. 1.0 (Ortiz 2018) to identify loci that likely contain co-assembled paralogs **[see**  **Supporting Information—Fig. S3****]**. In addition, we removed markers with more than 50 % missing data and, subsequently, individuals with more than 80 % missing data **[see**  **Supporting Information—Table S1****]**. Final VCF files were modified for different downstream population genomics analysis using vcfR v. 1.0.5 (Knaus and Grünwald 2017).

To test for reference genome bias against var. *filipes* samples, we mapped reads from var. *filipes* to the var. *filipes* reference genome (*P. hallii* var. *filipes* v3.1 v. at DOE-JGI, http://phytozome.jgi.doe.gov/) and found that the number of SNPs is very similar **[see**  **Supporting Information—Table S2****]** and phylogenetic trees are concordant when mapping to either genome **[see**  **Supporting Information—Fig. S4****]**.

### Species assignment via Sanger sequencing

As the taxonomic identification of species from *Panicum* section *Diffusa* is notoriously difficult (Waller 1976), we sequenced two chloroplast and one nuclear region to aid in taxonomic confirmation. Samples of *Panicum* section *Diffusa* species determined by taxonomic specialists at the Billie L. Turner Plant Resources Center (TEX) at The University of Texas at Austin were used as a control for species identification. A total of 31 samples were sequenced for the nuclear Internal Transcribed Spacer (ITS1) along with two new developed suitable chloroplast regions. The first marker, cp45676, spans the 3′ end of the intergenic region between *ycf3* and *trnS* and includes the 5′ portion of *trnS*. The second marker, cp1204, spans the 3′ end of the intergenic region between *psbA* and *trnK* and includes the 5′ portion of *trnK*. These markers were specially designed to differentiate species from the section *Diffusa* of the genus *Panicum*. The ingroup contained 17 control samples belonging to 11 species of the genus *Panicum*, section *Diffusa*. Also, six samples acted as known controls for both varieties of *P. hallii*. A sample of *Setaria viridis* was used as an outgroup for phylogenetic inference.

Approximately 570 bp of ITS1, 650 bp of cp1274 and 550 bp of cp45676 were amplified by polymerase chain reaction (PCR) using the same DNA samples that were sent for ddRAD. For ITS1, we used newly designed primers ddITS1F (5′-CCG TGA ACG TGT CAT CCA TG-3′) and ddITS1R (5′-GGT CCG AGC ACC AAG GCG-3′). For cp1204: 1204F (5′-GGC TTG TAC TTT CGC GTC TC-3′) and 1204R (5′-CGG TAC GAA CTT TTA TGC AAC G-3′). For cp45676: 45676F (5′-TAG GCA TAA TTC CCA ACC CA-3′) and 45676R (5′-CGA ACC CTC GGT AAA CAA AA-3′). Polymerase chain reaction mixtures were in 15 µL and were run on the MJR PTC-200 thermocycler. For ITS, we used the following conditions: 35 cycles of 95 °C denaturation for 60 s, 56 °C annealing for 45 s and 72 °C extension for 60 s. For the cp1204 and cp45676, we used a two-step PCR with 20 cycles of 95 °C for 60 s, 54 °C for 30 s, reduced by 0.1 °C every cycle, and 72 °C for 60 s, followed by 20 cycles of the same conditions but with a constant annealing temperature of 52 °C. Polymerase chain reaction amplicons were visualized with SYBRSafe (Invitrogen, Eugene, OR, USA) on 1 % agarose gels and prepared for cycle sequencing by treatment with a 10:1 mixture of Shrimp Alkaline Phosphatase and Exonuclease I (both from USB; Cleveland, OH, USA). Cleaned PCR products were submitted to The University of Texas at Austin ICMB Core Facility for cycle sequencing and visualization. The chromatograms were inspected by eye, manually trimmed for quality and aligned, then, the sequences were manually assembled, using Geneious v. 7.1.9 (Kearse *et al.* 2012). A resulting alignment of 1982 nucleotides was analysed, using Bayesian phylogenetic inference as implemented in MrBayes v. 3.2.2. The resultant trees were compared, exported and edited, using FigTree v. 1.4.3 (http://tree.bio.ed.ac.uk/software/figtree/). In addition, because the species in the *Diffusa* section also differ in ploidy, the ploidy level was estimated in these samples using flow cytometry analysis (BD LSRFostesca SORP).

### Population genomic structure

We calculated expected heterozygosity for all samples using VCFtools (--het) (Danecek *et al.* 2011). As a result of preliminary analyses, we selected San Antonio, TX as a landmark geographic location as it exhibited the maximum diversity of *P. hallii* in our sample. We calculated the distance of all samples to this landmark using the R package ‘geosphere’ v1.5-10 (Hijmans *et al.* 2019). We used the R function cor.test (R Core Team 2018) to test for correlation between expected heterozygosity and distance to San Antonio, TX for the var. *hallii* gene pools; the var. *filipes* gene pools lacked adequate sample sizes for the test.

### Analyses of phylogeographic structure

#### Phylogenetic estimation.

Relationships between *Panicum* species and the *P. hallii* varieties were assessed by performing maximum likelihood analyses implemented in IQ-TREE v. 1.5.5 (Nguyen *et al.* 2015). Because only variable sites were analysed, we used the model GTR+R+ASC to compensate for ascertainment bias; to provide nodal support we used the ultrafast bootstrap (Minh *et al.* 2013) implemented in IQ-TREE (Option –bb) with 10 000 pseudo-replicates.

#### Principal component analysis.

The high-quality SNP data set was used to identify *de novo* the optimal number of clusters and their relationship using discriminant analysis of principal component (DAPC) implemented in the Adegenet v. 2.1.1 package (Jombart 2008) for the R software (R Core Team 2018). Discriminant analysis of principal component centres on a discriminant function to maximize the differences between groups and minimizing variation within clusters (Jombart 2008). We ran the *k*-means clustering (find.clusters) to assess groups using the Bayesian information criterion (BIC); we used the cross-validation test (xvalDapc) to retain the number of PCs with the lowest mean squared error using 100 replicates. We used three different SNP data matrices for several independent analyses including the full set of 16 397 SNPs for all 423 samples of *P. hallii* species, one set of 16 595 SNPs for var. *hallii* and one set of 13 167 for var. *filipes*.

### Phenotypic divergence between *Panicum hallii* varieties

To explore the phenotypic divergence between var. *hallii* and var. *filipes*, a common garden was established on 15 May 2014. Four hundred and eighty individuals from 76 different localities (ranging from 1 to 22 individuals per locality) were planted at Pickle Research Campus, The University of Texas at Austin, TX, USA (latitude 30.3838 N, longitude −97.9296 W), following the seedling method described at Lowry *et al.* (2012). The experimental planting design followed a honeycomb layout with an interplant distance of 0.85 m. In total, seven phenotypic traits were chosen to evaluate divergence between the *P. hallii* varieties. During the months of July and August of 2014, one plant for each sampled locality was chosen to measure seed mass and panicle structure. For seed mass, 20 seeds per individual were weighed. Two representative mature panicles were collected and photographed to measure primary and secondary branch number and panicle length, using the Panicle Phenotyping tool P-trap (AL-Tam *et al.* 2013). On 29 August 2014, all samples were tested for cold tolerance using the electrolyte leakage method (Campitelli *et al.* 2013; Pérez-Harguindeguy *et al.* 2013) in leaves after a −3 °C frozen treatment. Early in Fall 2014, we harvested all plants and obtained above-ground biomass after drying samples for 1 week in a 65 °C oven. Finally, for 368 days after planting (between 15 May 2014 and 18 May 2015), we measured leaf senescence every 2 weeks for each plant, using a qualitative scale from 1 to 4 (1 > 50 % of leaf canopy green, 2 > 50 % of leaf canopy senesced, 3 > 90 % of leaf canopy senesced and 4 complete canopy senescence). The average quality state was taken as the output for each plant across the year.

### Correlation between genetic and phenotypic distances

A principal component analysis (PCA) was performed on the seven phenotypic traits using R v3.6.3 (R Core Team 2018) to identify the major axes of variation. We used the first two components from this analysis to build a matrix of pairwise phenotypic distances between populations. Similarly, we built a matrix of pairwise genetic differentiation between populations using the two first linear discriminants components (LD1 and LD2) from the DAPC analyses. Finally, a matrix of pairwise geographic distances between populations was constructed using the R package ‘geosphere’ v1.5-10 (Hijmans *et al.* 2019).

To test correlations between any two of these matrices, we performed a Mantel tests using the R package ‘vegan’ v2.5-6 (Oksanen *et al.* 2019) as well as a Partial Mantel test to evaluate associations between genomic and phenotypic distances while controlling by geographic distance. Mantel tests were performed using the Spearman correlation method and 9999 permutations.

### Admixture evaluation between *Panicum hallii* varieties

Genotypic and phenotypic analyses were conducted to explore the relationship between var. *filipes* and var. *hallii* in their region of sympatry. At the genotypic level, a new matrix of SNP was made including all var. *filipes* samples (27 individuals from 14 localities), samples from var. *hallii* in sympatry with var. *filipes* (24 individuals from 14 localities) and a sample of var. *hallii* samples allopatric to var. *filipes* (26 individuals from 12 localities). We assigned the allopatric var. *hallii* samples to one population, HAL, and a random subset of var. *filipes* samples to another population, FIL. We then ran STRUCTURE (Pritchard *et al.* 2000) using the USEPOPINFO model parameter and the ‘HAL’ and ‘FIL’ population assignments for 12 runs. The USEPOINFO model parameter included in the program STRUCTURE (Pritchard *et al.* 2000) was used to detect putative admixture individuals. In this approach, samples from known varieties are defined as ‘learning samples’ (PopFlag=1), and STRUCTURE attempts to group the unknown samples (PopFlag=0) to assign the membership probability associated with each variety (Porras-Hurtado *et al.* 2013). For the phenotypic analysis, a PCA was conducted with the ‘stats’ package in R v3.6.3 (R Core Team 2018), using the phenotypic data set obtained for the common gardens, but samples of var. *hallii* were divided into allopatric and sympatric samples.

## Results

### Sampling

**Figure 2.**
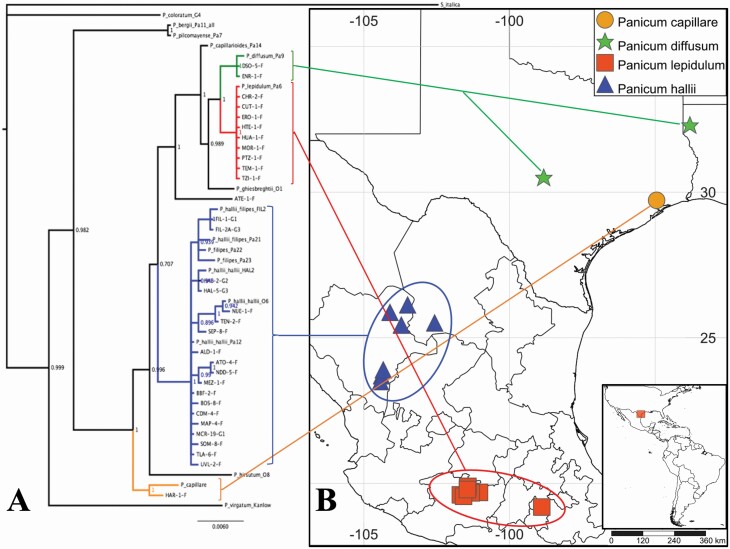
Species validation using nuclear and chloroplastics markers. (A) Bayesian phylogenetic analyses of the ITS nuclear marker and two chloroplast regions distinguish the different species from within the *Panicum* section *diffusa* (branch support values are a consensus from 1000 bootstrap replicates). Green lines indicate *P. diffusum* samples. Red lines indicate *P. lepidulum* samples. Blue lines indicate *P. hallii* collections. Orange clade indicates *P. capillare* collections. (B) Geographical distribution of the species collected.

This study included a total of 649 individuals primarily representing *P. hallii*. The sampling covered the entire geographic range of *P. hallii*, including collections from previously unsampled Mexican states and Texas counties (Fig. 1; Table 1). From these field collections, 28 localities were represented by a single individual, but most localities were represented by 2–22 individuals (4.5 individuals in average). Species identification for undetermined samples was completed using nuclear and plastid markers. Bayesian phylogenetic trees constructed with nuclear and plastid sequences clearly grouped with strong support our new collections against the control samples, confirming that the vast majority of our collected samples included three species belonging to the *Diffusa* section (Fig. 2). While the presence of *P. hallii* in Louisiana was recently reported (Reid and Urbatsch 2012), our sample from this state corresponds to *P. diffusum* not *P. hallii*. One collection in Central Texas was also assigned to *P. diffusum*, despite resembling *P. hallii* in the field. All samples collected in the central part of Mexico in the states of Mexico and Michoacan formed a clade with the *P. lepidulum* control sample, which had been collected in the same area. All samples from northern Mexico, from the states of Durango and Coahuila, clustered with *P. hallii.* Lastly, one sample in the eastern edge of the *P. hallii* was *P. capillare*. In addition, to the phylogenic analysis, the ploidy level measured with flow cytometry showed the polyploid status of the undetermined samples (*P. lepidulum* and *P. diffusum* are putatively 4x tetraploids), in contrast with the diploid condition of *P. hallii*  **[see**  **Supporting Information—Table S3****]**. We limited our subsequent analyses to 600 samples which had a strong phylogenetic assignment to *P. hallii*.

### ddRAD sequencing

We used the ddRAD approach to genotype the *P. hallii* samples. Almost all (99 %) of the raw reads passed our quality-filtering criteria. These high-quality reads were mapped against the *P. hallii* v. 2.1 reference genome assembly (DOE-JGI, http://phytozome.jgi.doe.gov/; Lovell *et al.* 2018). After genotyping, SNPs were filtered to cope with presumed paralogs and missing data at the markers and individual levels **[see**  **Supporting Information—Fig. S3****]**. Finally, after quality control steps, a matrix with 16 397 SNPs from 423 individuals of *P. hallii* belonging to 127 localities was obtained. In addition, individual SNP matrices were made for each variety with 16 595 SNPs for var. *hallii* (396 samples from 104 localities) and 13 167 for var. *filipes* (27 individuals from 14 localities). The high-confidence SNPs were fairly evenly distributed across the nine nuclear chromosomes of *P. hallii*  **[see**  **Supporting Information—Fig. S3C****]**.

### Natural distribution of *Panicum hallii* and HSM

Our sampling included regions where *P. hallii* has been described but for which contemporary samples are lacking. Several collection records show the presence of *P. hallii* samples in eastern Texas and Louisiana (Fig. 1A). However, after extensive field collection trips (16 localities), the only samples we collected were either *P. diffusum* or *P. capillare*. The absence of *P. hallii* from the East Central Texas forest, Western Gulf Coastal Grasslands and Pine Wood forest could indicate ecological constraints for establishment and persistence at the eastern edge of its distribution (Fig. 1B). A similar situation was found at the southern part of the natural distribution, where extensive sampling was conducted in the central states of Mexico, including the states of Mexico and Michoacan. However, both morphology and molecular markers indicate that collections made in 11 localities are *P. lepidulum* (Fig. 2). With these findings, the southern distribution of *P. hallii* seems to be restricted to the north of Mexico in the Meseta Central Matorral ecoregion.

Projections of the HSMs to the current climatic conditions encompass most of the known localities with high suitability scores. As expected, the areas with highest suitability match with the region of highest occurrence density located in South Texas. The consensus HSM for var. *filipes* includes the predictions of 134 (out of 175) independent models that showed true skill statistic (TSS) values above 0.75. The mean TSS over the full set of models was 0.83 and the area under the curve (AUC) score of 0.92 **[see**  **Supporting Information—Fig. S5****]**. Among the selected climate predictors, the mean temperature of warmest quarter was estimated as the most important variable to predict the presence of var. *filipes*  **[see**  **Supporting Information—Figs S6**  **and**  **S7****]**. The ensemble model for var. *filipes* shows high suitable areas from South Texas in the USA to the Nuevo Leon and Tamaulipas states in Mexico with decreasing suitability towards the south (Fig. 1; **see**  **Supporting Information—Fig. S8**). Also, distribution models show a clear limit to the west in the foothills of the Sierra Madre Oriental.

The consensus HSM for var. *hallii* includes the predictions of 134 (out of 175) independent models that showed TSS values above 0.75. The mean TSS over the full set of models was 0.78 and the AUC 0.94 **[see**  **Supporting Information—Fig. S9****]**. Among the selected climate predictors, temperature seasonality was estimated as the most important variable to predict the presence of *P. hallii*  **[see**  **Supporting Information—Figs S10**  **and**  **S11****]**. The projected suitable distribution for var. *hallii* shows a bigger continuous area with high suitability values encompassing the region of Texas and the states of Coahuila and Nuevo Leon in Mexico (Fig. 1; **see**  **Supporting Information—Fig. S12**). The projection for var. *hallii* also shows a nearly isolated suitable area in the far south-west between Arizona and New Mexico states in the USA.

The projections to past climatic scenarios of the HSMs for both varieties show similar distribution patterns for the mid-Holocene period compared to current climate and high agreement between the results from the three GCMs **[see**  **Supporting Information—Figs S8**  **and**  **S12****]**. Conversely, the projection to LGM and LIG periods shows a consistent southward shift and fragmentation in the distribution of suitable areas. Specifically, the predicted past distribution of var. *filipes* shows a strong reduction to scattered areas in the coast of the east of the Gulf of Mexico **[see**  **Supporting Information—Fig. S8****]**. Interestingly, there was almost no agreement among the projections obtained from the three GCMs for the LGM suggesting that this result should be interpreted with caution. For var. *hallii*, we found a well-supported suitable region that remains constant along all projected climatic scenarios in the northeast of Mexico **[see**  **Supporting Information—Fig. S12****]**.

### Divergence between *Panicum hallii* var. *hallii* and *Panicum hallii* var. *filipes*

**Figure 3.**
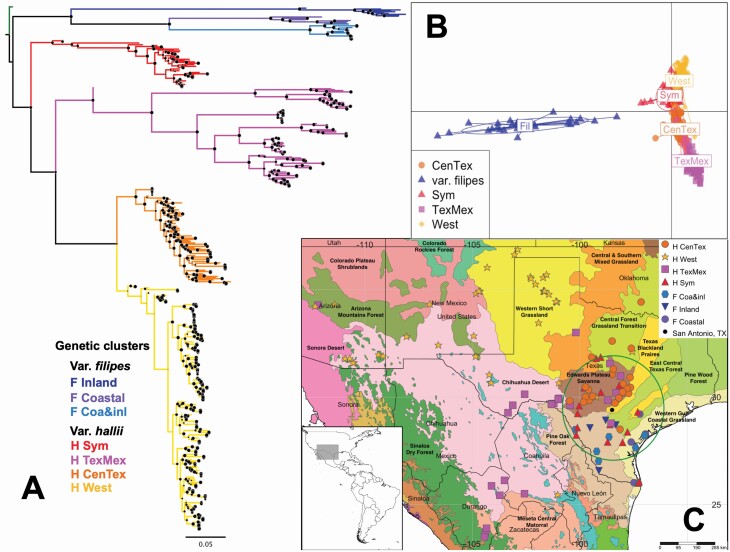
Geographic and genetic structure of 423 samples of *Panicum hallii*. (A) Maximum likelihood phylogenetic tree, with the blue cluster representing *P. hallii* var. *filipes* and the red, magenta, orange and yellow clusters representing *P. hallii* var. *hallii*. *Panicum virgatum* was used as an outgroup (size of black dots on the nodes is proportional to the node support derived from 10 000 ultrafast bootstrap). (B) Discriminant analysis of principal components (DAPC). (C) Geographical representation of the phylogenetic tree and DAPC analysis using an ecoregion map. The green circle represents the *P. hallii* diversity hotspot 200 km around San Antonio, TX. Maps produced using *SimpleMappr* (Shorthouse 2018).

Together, DAPC and phylogenetic analyses revealed substantial divergence between the *P. hallii* varieties as well as population structure within the varieties (Fig. 3). Phylogenetic analyses confirmed that each variety of *P. hallii* forms a monophyletic group (Fig. 3A). Summary statistics show that var. *filipes* has greater diversity (*H*_e_) than var. *hallii*, despite its narrow natural distribution (Table 1). Additionally, a Mantel test between DAPC genetic differentiation among populations and their corresponding geographic distances was highly significant (*P*-value < 0.0001) and had a positive Mantel statistic (*r* = 0.36) (Table 5; **see**  **Supporting Information—Fig. S13A**), supporting an isolation-by-distance pattern for both varieties.

### Population structure in *Panicum hallii* var. *hallii*

To examine the population structure of var. *hallii*, we investigated a set of 16 595 SNPs in 396 individuals from 104 localities. Using clustering methods, four groups were revealed by both the maximum likelihood tree and DAPC. The four genetic clusters present a clear geographic pattern. There is a large cluster in the South-western USA (Arizona, New Mexico and West Texas), hereafter called the ‘West’ cluster. In addition, there is a cluster including the Mexican samples and some Texas samples collected close to the Mexican border, identified as the ‘Tex-Mex’ cluster. In Central Texas, samples collected in the Edwards plateau and the Central Forest Grassland transition ecoregions form a group that we call the ‘Central Texas’ cluster. Finally, we identify a cluster occurring in South Texas where both varieties coexist under sympatric conditions that we name the ‘sympatric’ cluster. One hypothesis is that this population structure is the result of a northward and westward expansion of the species range following the LGM. To examine this hypothesis in var. *hallii*, we examined the correlation between expected heterozygosity and geographic distance to San Antonio. We expect a decreased genetic diversity as the distance from modern San Antonio (i.e. genetic diversity hotspot) increases. The West cluster shows a significant negative correlation between expected heterozygosity and distance from the hotspot. The other three clusters also show a negative, albeit non-significant, correlation between expected heterozygosity and distance to San Antonio (Table 2).

**Table 2.** Correlation between geographical distance and genetic diversity (*H*_e_, heterozygosity) in *Panicum hallii* variety *hallii* genetic clusters from the diversity hotspot.

Genetic cluster	Number of samples	Correlation	*P*-value
Central Texas	90	−0.1377	0.1954
West of the distribution	187	−0.3568	0.00000055
Sympatry area	32	−0.2335	0.1982
Texas-Mexico	87	−0.0722	0.5060

**Figure 4.**
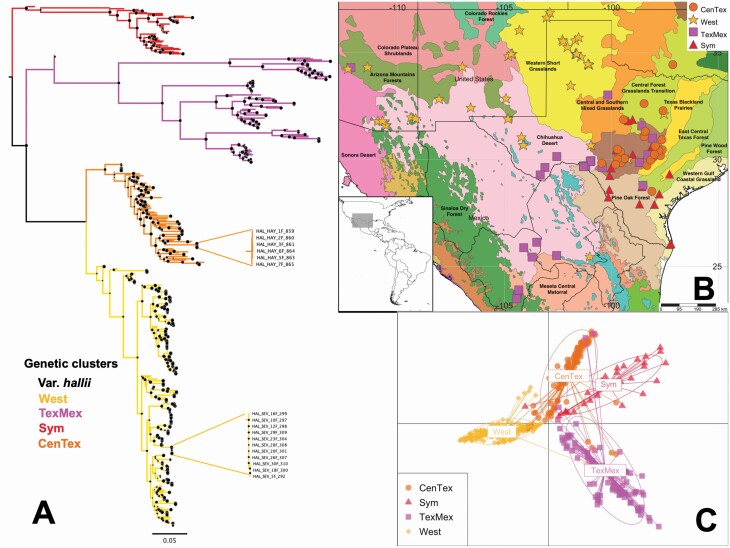
Geographic and genetic structure of 396 samples of *Panicum hallii* var. *hallii* from 104 localities. (A) Maximum likelihood phylogenetic tree rooted according to Fig. 3, including inset clades depicting the close relationship between individuals from single collection locations (size of black dots on the nodes is proportional to the node support derived from 10 000 ultrafast bootstrap). (B) Geographical and ecological representation of the clustering analysis. (C) Discriminant analysis of principal components.

In addition to the broad geographic structure of the genetic diversity in var. *hallii*, individuals collected in each locality usually group together as a monophyletic group in the phylogenetic tree (Fig. 4A).

### Population structure in *Panicum hallii* var. *filipes*

**Figure 5.**
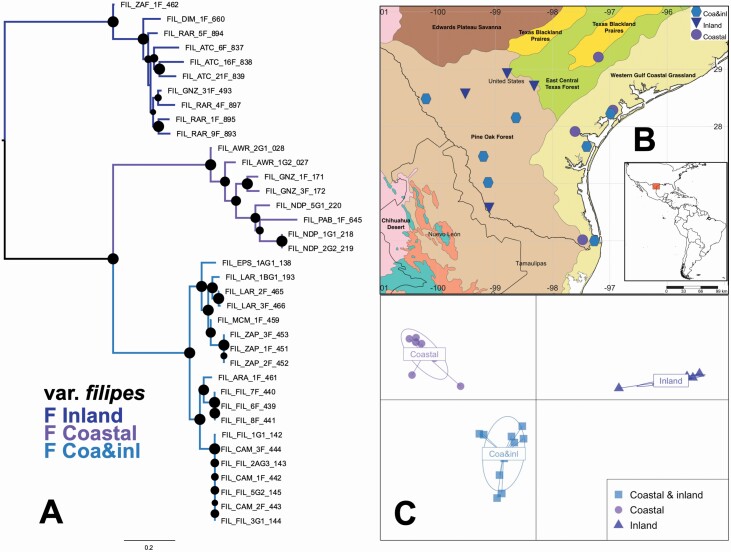
Geographic and genetic structure of 27 samples of *Panicum hallii* var. *filipes* from 14 localities. (A) Maximum likelihood phylogenetic tree rooted according to Fig. 3 (size of black dots on the nodes is proportional to the node support derived from 10 000 ultrafast bootstrap). (B) Geographical and ecological representation of the clustering analysis. (C) Discriminant analysis of principal components.

Despite the narrow distribution of var. *filipes* in South Texas, this variety shows strong population structure divided into three clusters (Fig. 5): one genetic cluster has a coastal distribution, another group has an inland distribution and a third widespread group with individuals collected in both inland (Pine Oak Forest ecoregion) and coastal areas (Western Gulf Coastal Grasslands ecoregion) (Fig. 5).

### Phenotypic divergence between *Panicum hallii* var. *filipes* and *Panicum hallii* var. *hallii*

**Figure 6.**
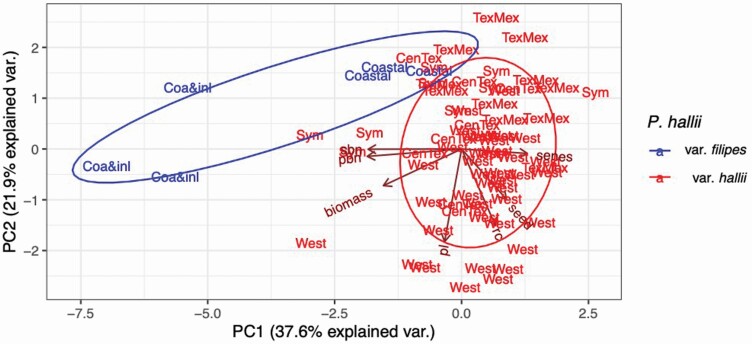
Principal component analysis of seven phenotypic *characters* in *Panicum hallii* var. *filipes* (blue) and var. *hallii* (red labels represent the genetic cluster).

Principal component analysis using seven phenotypic characters showed a high divergence between var. *filipes* and var. *hallii* (Fig. 6). In general, var. *filipes* had larger morphological features (excepting seeds) than var. *hallii* (Table 3). Also, var. *filipes* was more tolerant to cold stress (measured as a percentage of electrolyte leakage). Lastly, the lifespan (measured as senescence score across time) was longer for var. *filipes* than var. *hallii*.

**Table 3.** Morphological traits mean (standard error) for *Panicum hallii* var. *filipes* and *Panicum hallii* var. *hallii* measured in two different seasons and three localities.

Trait	var. *filipes*	var. *hallii*
Panicle length (cm)	24.75 (2.44)	26.38 (5.22)
Primary branch number	13.83 (4.05)	9.72 (1.5)
Secondary branch number	45.25 (28.39)	17.31 (9.10)
Biomass (g)	247.76 (82.28)	151.92 (52.83)
Senescence score	1.81 (0.16)	2.43 (0.27)
% electrolyte leakage	15.67 (9.67)	33.89 (12.39)
Seed mass (mg)	0.77 (0.18)	1.41 (0.65)

The seven phenotypic characters also illustrate phenotypic divergence between genetically distinct geographic clusters (Fig. 6). With PCA, we see clustering differences between geographic regions, especially in var. *filipes*, where the samples collected in the coastal and inland form two different clusters. Interestingly, the var. *hallii* samples belonging to the sympatric group form a morphology-based cluster that is intermediate between the var. *filipes* and the rest of the var. *hallii* samples. Our Mantel test revealed a positive correlation between phenotypic distance of populations and their geographic distance (*r*s = 0.23, *P* < 0.001; Table 5; **see**  **Supporting Information—Fig. S13B**). Thus, confirming our field observations of increased morphological differentiation as a function of geographic distance. Similarly, we also found support for the correlation between the DAPC genetic distances and the PCA phenotypic distances supported by a Mantel test (*r* = 0.24, *P*-value = 0.0005; Table 5; **see**  **Supporting Information—Fig. S13C**). This correlation held even after controlling by geographic distance in a Partial Mantel test (*r* = 0.17, *P*-value = 0.0094) (Table 5).

### Genotypic and phenotypic analyses in the sympatric area

In South-west Texas, where var. *filipes* and var. *hallii* coexist in sympatry, some samples appear intermediate between the two varieties when genotypes and phenotypes are clustered (Fig. 6). Though *P. hallii* is a highly self-fertilizing species (Lowry *et al.* 2012, 2013), the two varieties can be crossed in the greenhouse (Lowry *et al.* 2015), and outcrossing in greenhouse conditions has been observed (X. Weng, pers. comm. Department of Integrative Biology, The University of Texas at Austin). To determine if intermediate samples resulted from gene flow between the two varieties, we generated a new matrix of 12 463 SNP from all samples collected in the sympatric region (27 var. *filipes* from 14 localities and 24 var. *hallii* from 14 localities) plus some var. *hallii* samples (26 individuals from 12 localities) collected in allopatric regions to serve as non-admixed controls.

After running the program STRUCTURE (Pritchard *et al.* 2000) to evaluate admixture between varieties, six individuals labelled as var. *hallii* collected from four localities in the sympatric zone were assigned membership to both var. *filipes* and var. *hallii* groups under four different arrangements of var. *filipes* learning samples (Table 4), suggesting that these samples are admixed and there is at least periodic gene flow between var. *hallii* and var. *filipes* in sympatry.

**Table 4.** Membership probability of inferred ancestry component for individuals of *Panicum hallii* var. *hallii* collected in the sympatric zone using four sets of *Panicum hallii* var. *filipes* learning samples.

	var. *filipes* learning samples set 1		var. *filipes* learning samples set 2		Removing var. *filipes* misclassified samples		All var. *filipes* as a learning samples	
Individual	FIL	HAL	FIL	HAL	FIL	HAL	FIL	HAL
BEE_1AG	0.55	0.45	0.49	0.51	0.35	0.65	0.57	0.43
BEE_3F	0.13	0.87	0.12	0.88	0.04	0.97	0.14	0.86
GNZ_22F	0.42	0.58	0.38	0.63	0.48	0.52	0.43	0.57
GNZ_24F	0.58	0.42	0.52	0.48	0.22	0.78	0.60	0.40
RAR_8F	0.33	0.67	0.28	0.72	0.03	0.97	0.33	0.67
UVL_1AG	0.27	0.73	0.24	0.76	0.57	0.43	0.28	0.72

Morphological analysis of the samples of var. *hallii* collected in the sympatry area showed intermediate phenotypic characteristics between samples of var. *filipes* and samples of var. *hallii* growing in allopatry. Phenotypic traits such as secondary branch number, biomass, tiller number, seed mass and senescence presented intermediate values between these two varieties. In addition, the PCA of the phenotypic data showed that the sympatric localities of var. *hallii* formed a group between the samples of var. *filipes* and var. *hallii* in allopatry. This intermediate behaviour is especially true for two (BEE.1A and UVL.1A) of the six samples with admixed compositions in the genotypic analysis **[see**  **Supporting Information—Fig. S14****]**.

## Discussion

### Genetic and morphological divergence between *Panicum hallii* varieties

We used ddRAD to genotype more than 400 *P. hallii* individuals across the natural range of the species. We detected strong genomic differentiation between the two varieties of *P. hallii*, as indicated by the phylogenetic tree (Fig. 3A) and the discriminant analysis (Fig. 3B). More generally, we found a strong signal of genetic divergence as a function of geographic distance as shown by a Mantel test (Table 5; **see**  **Supporting Information—Fig. S13A**). This was consistent with the genetic architecture of differentiation detected with QTL (Lowry *et al.* 2015) and morphology (Waller 1976; Lowry *et al.* 2013). The two varieties are typically found in contrasting habitats, with var. *hallii* growing in xeric habitats and var. *filipes* found in more mesic habitats. This habitat preference was observed in the field and confirmed with our collections. Indeed, Gould *et al.* (2018) found that climatic variables related with extreme conditions such as minimum temperature and temperature daily range are key variables correlated with individual genetic distances in *P. hallii* as a whole. The most important climatic predictors estimated for the HSM corroborate that variables representing temperature variation through the year (e.g. temp. annual range for var. *filipes* and temp. seasonality for var. *hallii*), extreme temperature conditions (e.g. mean temp. of warmest quarter for var. *filipes* and mean temp. of wettest quarter for var. *hallii*) are the better predictors of the *P. hallii* varieties distribution. We also corroborate annual precipitation as important predictor for var. *hallii* which almost occupies the geographic range of the entire species, but not for var. *filipes* that is more restricted to coastal and foothill environments and should be less tolerant to low precipitation conditions **[see**  **Supporting Information—Figs S7**  **and**  **S11****]**.

**Table 5.** Mantel test statistics. *Partial Mantel test using geographic distance as a controlling variable.

Test	Mantel’s *r*	*P*-value
Genotypic DAPC ~ geographic	0.3595504	0.0001
Phenotypic PCA ~ geographic	0.2339050	0.0002
Phenotypic PCA ~ genotypic DAPC	0.2363396	0.0005
Phenotypic PCA ~ genotypic DAPC | geographic*	0.1678044	0.0094

We also detected morphological differentiation between the varieties, particularly in traits that may confer ecological adaptation. For instance, var. *hallii* has heavier seeds than var. *filipes*  **[****Supporting Information—Fig. S14****]**, which is a common pattern for plants that are typically exposed to drought conditions after germination in their natural habitat (Baker 1972). We also detected differences in cold tolerance between the varieties **[see**  **Supporting Information—Fig. S14****]**, with the var. *hallii* showing greater cold sensitivity than the var. *filipes*. This was surprising, given that var. *hallii* inhabits colder regions and higher altitudes than var. *filipes*. In addition, although *P. hallii* has been described as a perennial grass, lifespan evaluations show that var. *filipes* has longer lifespan than var. *hallii* under controlled common garden conditions (**see**  **Supporting Information—Fig. S14**, senescence). Short lifespan in var. *hallii* might be associated with other life history characters important for plant survival in dry environments, such as early flowering time, summer dormancy and bigger seed mass (Lowry *et al.* 2012, 2013). In combination, these traits may allow var. *hallii* to rapidly complete its life cycle during the short-wet seasons and avoid the stresses of the dry seasons and winters.

Interestingly, the morphological differentiation pattern was found between and within varieties as indicated by the Partial Mantel tests (Table 5; **see**  **Supporting Information—Fig. S13B**  **and**  **C**) which show a significant positive correlation between phenotypic distance and geographic distance as well as between phenotypic and genetic distance (which remains highly significant and positive even after controlling by geographic distance in a Partial Mantel test; Table 5). An isolation-by-distance pattern can be deduced from these correlations, where we can observe that as the geographic distance increases between populations, so does genetic divergence and the concomitant phenotypic separation.

Across much of the range of *P. hallii*, there are strong geographic and ecological barriers to gene flow which likely promote and/or reinforce differentiation between the varieties. However, in southeast Texas, var. *filipes* and var. *hallii* grow in sympatry and are subject to similar climate conditions. Climate-related selection pressures promoting phenotypic differentiation may be relaxed, because var. *hallii* samples from this sympatric region are less morphologically differentiated from var. *filipes* than they are from var. *filipes* from allopatric regions **[see**  **Supporting Information—Fig. S14****]**. In this sympatric area, where geographic and ecological barriers to gene flow are weakened, we detect evidence of admixture between the varieties in at least six samples from four localities. Our results suggest the existence of inter-mating between *P. hallii* varieties despite an estimated divergence of ~1 M years (Lovell *et al.* 2018). For instance, in the eastern locality close to Gonzales, TX, genetic analysis of nine samples showed that individuals from both varieties are present at the same location (one for var. *filipes* and six var. *hallii*). Two samples from that site appear to be admixed, suggesting natural crosses of these two varieties. The Gonzales locality is particularly notable because the morphological and ecological characteristics are not typical for *P. hallii*. Morphologically, we were not able to definitively differentiate the two varieties in the field at this locality. Ecologically, this is the only locality in the Texas Blackland Prairie ecoregion where we collected both varieties of *P. hallii*, despite extensive collecting effort at other localities in the ecoregion. However, while there is detectable hybridization in the Gonzales locality, samples of both varieties with no evidence of admixture are also found in this locality, indicating that hybridization is not ubiquitous. Potential reproductive barriers between the two varieties have yet to be quantified, as they have been for other plant species (Lowry *et al.* 2008; Sobel and Chen 2014). However, Lowry *et al.* (2015) did identify a two-locus Dobzhansky–Muller hybrid incompatibility causing sterility in a cross between var. *hallii* and var. *filipes*, which suggests that there is intrinsic postzygotic isolation between the two varieties.

### Population structure within varieties

The two varieties of *P. hallii* are adapted to different environments, but we observe evidence of considerable differentiation within each variety as well. In a broad sense, var. *hallii* has a geographical break at the genetic structure level in West Texas, coinciding with the Upper Cretaceous sediment to the West and most recently Paleogene, Neogene and Quaternary deposits to the East around the 100° W meridian. This break isolates the West and Tex-Mex groups from the other genetic clusters. This geological boundary suggests that the type of soil can also explain the genetic pattern found in var. *hallii*. To the east of this geological boundary is the Central Texas group, which is almost exclusively restricted to the Edwards Plateau and Central forest–grassland transition ecoregions, suggesting that there are either physical or ecological barriers constraining this group to these ecoregions. Within var. *filipes*, the coastal and inland genetic groups are found only in the Western Gulf Coastal Grasslands and Pine Oak Forest ecoregions, respectively, suggesting that their differentiation is related to ecological differences between those regions. Interestingly, the third var. *filipes* group, which is found in both Western Gulf Coastal Grasslands and Pine Oak Forest ecoregions, has the most divergent morphological traits of any group and has a clear perennial lifespan, suggesting that the differentiation of this group is related to life history strategy. This relationship between population structure and ecoregions suggests that adaptive differences play a major role in shaping genetic diversity not only between varieties but also within varieties of *P. hallii*.

### New insights of the species historical demography

There is strong population genetic structure within *P. hallii*, with two divergent intraspecific lineages already classified as varieties, and a total of seven genetic clusters. A diversity hotspot was found in southern Texas, where at least one representative of each genetic cluster occurs within a radius of 200 km from San Antonio, TX (Fig. 3). There are at least three non-exclusive reasons why this region is the centre of genetic diversity for *P. hallii*: diverse ecological conditions, complex demographic processes in response to the Quaternary climatic fluctuations and potential admixture between the varieties. Given that the projections of the HSMs to past climate scenarios do not support San Antonio area as a climatic stable region, we propose a scenario of strong distribution shifts and fragmentation but with mild bottlenecks and that the high diversity in the San Antonio region is a joint product of post-glacial recolonization, high recent ecological diversification and hybridization. Below, we discuss the evidence supporting this plausible scenario.

From the ecological perspective, the ecological heterogeneity of Central Texas can be a key promoter of diversification. This region belongs to the recently proposed North American Coastal Plain (NACP) global biodiversity hotspot (Noss *et al.* 2015), characterized by its high endemism of vascular plants, especially in its grassland coastal biomes (MacRoberts *et al.* 2002; Noss 2014). Moreover, this area contains seven ecoregions, defined as a land area containing a particular assembly of natural communities and species (Olson *et al.* 2001) (Fig. 1). In addition, at the plant diversity level, a mix of three floristic provinces coincides in Central and South Texas, where around 20 % of the endemic plants are grasses (Sorrie and Weakley 2001). Specifically, the area around San Antonio, TX, contains five grassland community types (Fayette, Upper Coastal, Coastal, Blackland and San Antonio Prairie) characterized by seven major soil associations (Diamond and Smeins 1985). Taken together, these ecological factors likely contribute to the high diversity of *P. hallii* by promoting multiple local adaptation processes over short time scales in this narrow area in Central Texas and the Rio Grande Valley.

It is well known that the Quaternary climatic fluctuations in the form of multiple contraction–expansion cycles of species distribution shaped the contemporary genetic composition of the biodiversity (Hewitt 2000, 2004; Razgour *et al.* 2013). Strong distribution shifts during the LGM as well as disagreement between the predicted distribution areas resulted from the projections using different GCMs were already reported in plants from South Texas (Rebernig *et al.* 2010). During the glacial cycles of the Pleistocene, South Texas was a glacial refugium belt for boreal displaced vegetation from the northern boreal latitudes (Holmgren *et al.* 2007) and for lowland taxa during the flooding due to sea level fluctuations (Noss *et al.* 2015). It served as a refugium for a variety of plants and animals promoting species richness and genetic diversity (Elias 1992; Swenson and Howard 2005; Majure *et al.* 2012; Bryson *et al.* 2014; Seal *et al.* 2015). A large-scale aridification of the south-western parts of North America started during the Holocene at the end of the LGM (~21 000 ybp), allowing the range expansion of drought-adapted genotypes from the refuge areas to the new arid regions (Rebernig *et al.* 2010) following the Northwest outward-direction, post-glacial expansion route proposed by Swenson and Howard (2005) for Central Texas refugia. An expansion of the var. *hallii* during Holocene might explain the lack of diversity in the recently colonized regions (i.e. West and Tex-Mex genetic clusters). In fact, there is a significant negative correlation between diversity (heterozygosity) and distance from southern Texas (San Antonio) for the var. *hallii* West cluster, consistent with a post-glacial expansion (Table 2). A similar pattern has been found in blackfoot daisy (*Melampodium leucanthum*, Asteraceae) (Stuessy *et al.* 2004; Rebernig *et al.* 2010) and members of the *Humifusa* clade of *Opuntia* genus (Majure *et al.* 2012).

Finally, diversity may be high in Central Texas due to hybridization between the varieties. During the Pleistocene, there were recurrent glacial cycles and the species range likely contracted and expanded multiple times. These successive contraction–expansion events led to the formation of contact zones, hybrid zones, and phylogeographic break areas in North America (Suture zones) (Remington 1968; Hewitt 2001), one of them in Central Texas (Swenson and Howard 2005, 2004), which correspond to the genetic diversity hotspot found for *P. hallii* in this study. Thus, the observed high diversity may be due to a history of secondary contact at this location between groups that diverged in allopatry during previous glacial cycles. More extensive sampling in the sympatric zone for both varieties and the use of plastid genomic information can be used to address this question.

### *Panicum hallii* distribution

The results from our field sampling suggested that the natural distribution of *P. hallii* is narrower than previously considered at the southern and eastern edges of the species distribution. Previous literature and collection records indicate that the natural distribution of *P. hallii* spans from the South-west USA (Oklahoma, Colorado, Texas, New Mexico and Arizona) through Mexico to the southern Mexican state of Oaxaca (Gould 1975; Waller 1976; Herrera and Pámanes 2006; Estrada and Villareal 2010; Herrera-Arrieta and Cortés-Ortiz 2010; Sánchez-Ken 2011; Valdés-Reyna *et al.* 2015). However, our collection efforts in the southern portion of this reported range, in the Mexican states of Mexico and Michoacán, failed to find *P. hallii*. The plants we found that most resemble *P. hallii* have morphological characteristics not found in *P. hallii* samples collected in the USA, and molecular markers indicate that these plants correspond to *P. lepidulum* (Fig. 2). We focused our Southern Mexican collection efforts in areas where herbarium samples were previously collected, and the absence of *P. hallii* in these Mexican states suggests that the southern boundary of the distribution of *P. hallii* is currently the Meseta Central Matorral and Chihuahua Desert ecoregions in the Northern Mexican states (Fig. 1B).

At the eastern part of the distribution, several collection records indicate that *P. hallii* grows in eastern Texas close to the Louisiana border and even in northwest Louisiana (Reid and Urbatsch 2012). However, our results showed that these individuals genetically grouped with two different *Panicum* species (*P. diffusum* and *P. capillare*). We also made at least 14 collection trips east of the Edwards Plateau ecoregion in Central Texas without finding *P. hallii*, suggesting that the eastern distribution of *P. hallii* is restricted to the Texas Blackland Prairies and East Central Texas forest ecoregions (Fig. 1B).

The projections of the HSMs for current conditions also support these proposed eastern and southern distribution limits (Fig. 1). Despite the fact that we trained our species distribution models including putative occurrences located beyond these limits (based on secondary reports), the distribution limits suggested by the HSM agree with the limits of the mentioned ecoregions and exclude doubtful records beyond these limits.

Overall, var. *hallii* is associated primarily with six ecoregions: the Meseta Central Matorral in the South, the Chihuahuan Desert and the Arizona Mountain Forest ecoregion to the West, the Western Short Grasslands and Central Forest Grasslands Transition to the North, and finally, the Central and Southern Mixed Grasslands and Edwards Plateau Savanna to the East (Fig. 1B). The distribution of var. *filipes* is restricted to the Western Gulf Coastal Grasslands and Pine Oak Forest ecoregions. The var. *hallii* was also collected in these ecoregions, but it was by far less abundant. According to the projections of the HSMs, the distribution of var. *filipes* encompasses a subset of the var. *hallii* distribution in its eastern range. Moreover, the habitat suitability model supports a conspicuous western limit near to the foothills of the Sierra Madre Oriental for the var. *filipes* (Fig. 1).

### Lessons for diversity studies in morphologically and ecologically diverse systems

One challenge we encountered while trying to sample diversity across the range of *P. hallii* was to correctly distinguish *P. hallii* from closely related species. *Panicum hallii* grows in a variety of habitats, and its morphology in natural and controlled settings can differ substantially. We collected 45 samples from the originally reported range of *P. hallii* that were members of other species in *Panicum* section *Diffusum*. There are many research systems that share this characteristic: ecologically and morphologically diverse taxa that are difficult to distinguish from closely related taxa in natural setting (Rebernig *et al.* 2010; Tyler *et al.* 2016). Sampling the breadth of genetic, morphological and ecological diversity is important, but can lead to the collection of non-target taxa. While including all collected samples in sequencing-based genotyping methods (e.g. ddRAD, GBS) can resolve the relationships among target and non-target taxa, that may not be the most efficient use of sequencing, computing or analysis resources. For instance, sequencing effort of the target taxa is decreased when non-target taxa are included. In addition, in our experience, methods for identifying SNPs and calling genotypes in non-model species can be sensitive to phylogenetic diversity (Mastretta-Yanes *et al.* 2015; Shafer *et al.* 2017), and starting strictly with the target taxa reduces the filtering steps necessary to produce a useful set of SNPs for subsequent analyses.

Therefore, we recommend that, if possible, samples be checked in a controlled environment in order to identify samples that do not match the standard description of the taxon, though we recognize this is not possible for many systems. Then a quick method should be used to verify that suspicious samples are within the intended taxonomic level for the study. For this study, Sanger sequencing of nuclear and chloroplast loci was fast and provided the resolution required to differentiate species. Finally, the next-generation sequencing (NGS) efforts should focus on the verified samples. While the initial steps take time and may delay NGS efforts, they are a valuable investment as they improve the sequencing results and can save substantial time during bioinformatic analysis.

In conclusion, the genetic and morphological evaluation in this study represents the most extensive sampling of the *P. hallii* varieties. This evaluation made it possible to distinguish genetic structure at different biological levels. At the species level, divergence was found between both varieties using molecular and morphological data. Within varieties, it was possible to characterize population structure across the diverse geological and ecological native range of *P. hallii*. Within the sympatric zone, this set of data allowed us to identify putative admixed individuals between var. *filipes* and var. *hallii*. Taken together, these data illustrate the geographical distribution of genetic diversity in *P. hallii*. A practical application of these results could be the creation of a core collection of these samples to streamline screening for particular traits of interest. Future work could include increased sampling efforts for var. *filipes*, especially in the Rio Grande Valley and Mexico to explore the genetic diversity in the Western and South boundary of the distribution. In addition, to confirm admixture between the varieties, the plastome from samples in the sympatric area can be examined to confirm admixture between the varieties. Furthermore, studies of local adaptation could be conducted by crossing members of different populations (Lowry *et al.* 2015; Milano *et al.* 2016), or by reciprocal transplants across ecoregions to identify the traits that underlie ecotype formation (Hereford 2009). Together, our data and analyses provide a foundation for understanding the natural history and evolution of *P. hallii* and highlight some of the important factors leading the observed structure and genetic diversity in the group.

## Supporting Information

The following additional information is available in the online version of this article—

Table S1. Sequence of matrix data-filtering process.

Table S2. Differences in SNP calling in *Panicum hallii* var. *filipes* when is using both available genomes, var. *hallii* v. 2.1 and var. *filipes* v. 3.1 before and after filtering processes.

Table S3. Ploidy level measure by flow cytometry profile from some *Panicum* section *Diffusum* species collected.

Figure S1. Whole plant morphology of *Panicum hallii* varieties.

Figure S2. Sampling points for presence, absence and pseudo-absence locations for *Panicum hallii* var. *filipes* and *P. hallii* var. *hallii*.

Figure S3. Paralogs filtering *Panicum hallii* var. *hallii* and *Panicum hallii* var. *filipes* data set.

Figure S4. Maximum likelihood phylogenic tree in *Panicum hallii* var. *filipes* using markers mapped against different genome references.

Figure S5. Boxplots for the individual model evaluation statistics area under the receiver operating characteristic curve (AUC) and the true skill statistic (TSS) for the habitat suitability modelling for *Panicum hallii* var. *filipes*.

Figure S6. Correlation matrix of bioclimatic selected variables for the habitat suitability modelling for *Panicum hallii* var. *filipes*.

Figure S7. Variable importance for the habitat suitability modelling for *Panicum hallii* var. *filipes*.

Figure S8. Projections of the ensemble habitat suitability model of *Panicum hallii* var. *filipes* to Last Inter-Glacial, Last Glacial Maximum, mid-Holocene and current climatic conditions.

Figure S9. Boxplots for the individual model evaluation statistics area under the receiver operating characteristic curve (AUC) and the true skill statistic (TSS) for the habitat suitability modelling for *Panicum hallii* var. *hallii*.

Figure S10. Correlation matrix of bioclimatic selected variables for the habitat suitability modelling for *Panicum hallii* var. *hallii*.

Figure S11. Variable importance for the habitat suitability modelling for *Panicum hallii* var. *hallii*.

Figure S12. Projections of the ensemble habitat suitability model of *Panicum hallii* var. *hallii* to Last Inter-Glacial, Last Glacial Maximum, mid-Holocene and current climatic conditions.

Figure S13. Mantel tests results.

Figure S14. Phenotypic divergence between and within varieties *Panicum hallii* var. *filipes* and *Panicum hallii* var. *hallii* and between *Panicum hallii* var. *hallii* regions.

plab002_suppl_Supplementary_File_1

plab002_suppl_Supplementary_File_2

## Data Availability

The raw reads were submitted to the National Center for Biotechnology Information (NCBI) Short Read Archive (SRA) under project number SRP110713.

## Notes

**Evolution & Diversity. Chief Editor:** Jeremy Beaulieu
