# Supplementary material for: Geographic patterns of genomic diversity and structure in the C4 grass Panicum hallii across its natural distribution
Source: AoB Plants. 2021 Jan 6;13(2):plab002. doi: 10.1093/aobpla/plab002 (PMC7937184; doi:10.1093/aobpla/plab002)
Supplement: plab002_suppl_Supplementary_File_1 [file plab002_suppl_supplementary_file_1.pdf]

Geographic patterns of genomic diversity and structure in the C_4 grass *Panicum hallii* across its natural distribution

Supplementary information

Supplementary tables

Table S1. Sequence of matrix data filtering process

Data set	Localities	Individuals	SNP
All samples	142	649	1,304,991
Panicum hallii	127	600	1,088,183
var. *filipes*	15	42	638,293
var. *hallii*	111	558	955,841
Panicum lepidulum	10	35	255,052
After remove paralogs			
Panicum hallii	127	591	258,151
var. *filipes*	15	37	47,951
var. *hallii*	554	112	217,157
After filtering by one SNP per stack			
Panicum hallii	127	591	45,589
var. *filipes*	15	37	40,816
var. *hallii*	554	112	45,146
After filtering by missing data			
Panicum hallii	118	423	16,397
var. *filipes*	14	27	13,167
var. *hallii*	104	396	16,595

Table S2. Differences in SNP calling in Panicum hallii var. filipes when is using both available genomes, var. hallii v. 2.1 and var. filipes v. 3.1 before and after filtering processes.

Reference genome	Filtering	SNPs
Panicum hallii var. hallii v. 2.1	Before remove paralogs	1,191,908
Panicum hallii var. filipes v. 3.1	Before remove paralogs	1,115,993
Panicum hallii var. hallii v. 2.1	After remove paralogs	8,514
Panicum hallii var. filipes v. 3.1	After remove paralogs	8,828

Table S3. Ploidy level measure by flow cytometry profile from some *Panicum* section *Diffusum* species collected

Species	Genotype	Ploidy	IP	Absolute DNA
Panicum hallii* var. *filipes	CAM.1.G1	2X	33903	0.54
Panicum hallii* var. *filipes	FIL.2	2X	34733	0.55
Panicum hallii* var. *filipes	FIL.2	2X	34368	0.54
Panicum hallii* var. *filipes	FIL.2	2X	28795	0.46
Panicum hallii* var. *filipes	FIL.2	2X	27611	0.44
Panicum hallii* var. *filipes	FIL.2	2X	30029	0.48
Panicum hallii* var. *filipes	FIL.2	2X	29797	0.47
Panicum hallii* var. *filipes	FIL.2	2X	26561	0.42
Panicum hallii* var. *hallii	MCR.19.G2	2X	37359	0.59
Panicum hallii* var. *hallii	HAL.2	2X	32020	0.51
Panicum hallii* var. *hallii	HAL.2	2X	31810	0.50
Panicum hallii* var. *hallii	HAL.2	2X	31783	0.50
Panicum hallii* var. *hallii	HAL.2	2X	32231	0.51
Panicum hallii* var. *hallii	HAL.2	2X	28393	0.45
**Panicum cf. lepidulum**	TEN.1.G1	4X	83392	1.33
**Panicum cf. caplillare**	HAR.29.F	4X	93630	1.49
**Panicum cf. diffusum**	ENR.4.F	4X	65811	1.05
**Panicum sp.**	ALD.1.G1	4X	87424	1.39
**Panicum sp.**	ALD.1.G1	4X	62545	1.00
**Panicum sp.**	ALD.1.G1	4X	60685	0.97

Supplementary figures

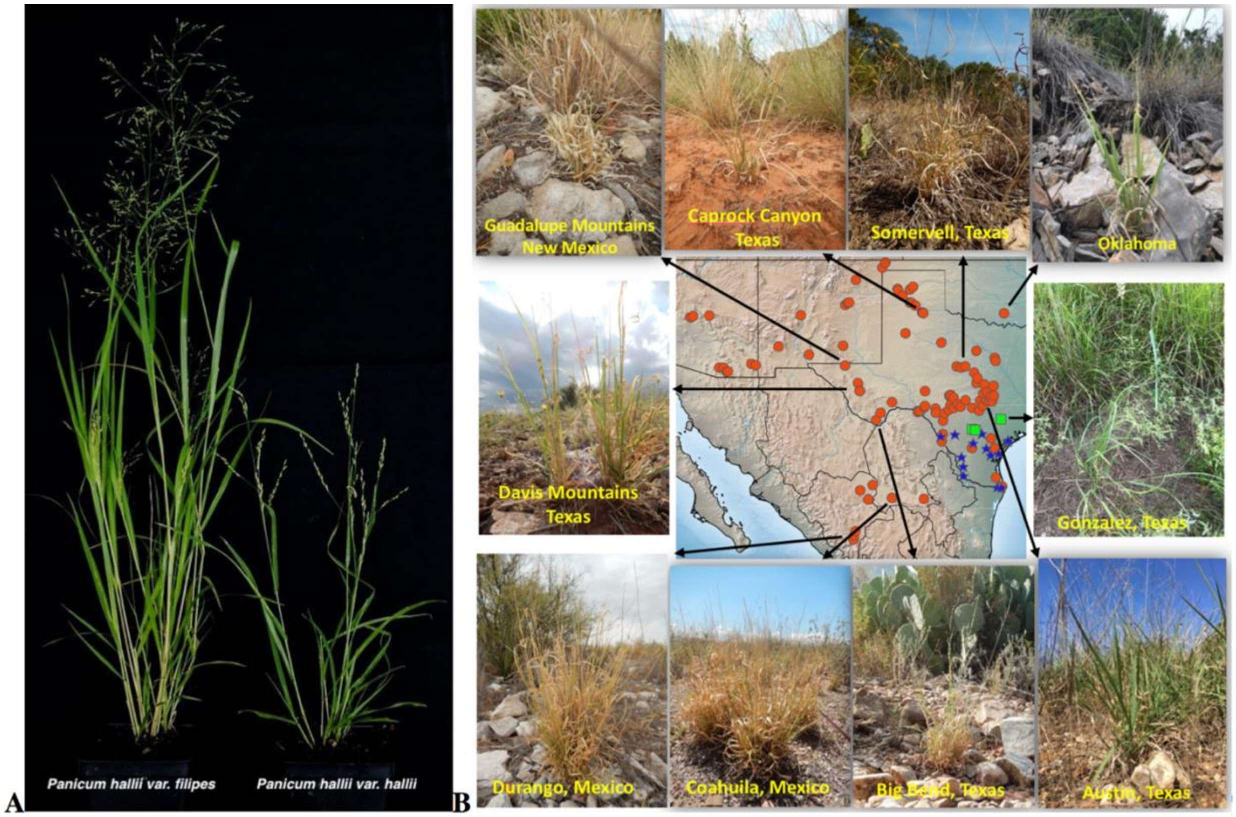

Figure S1. Whole plant morphology of *Panicum hallii* varieties. A. Differences between *Panicum hallii* var. *filipes* (Left) and *Panicum hallii* var. *hallii* (Right). B. Whole plant aspect of *Panicum hallii* var. *hallii* across its geographical range of distribution.

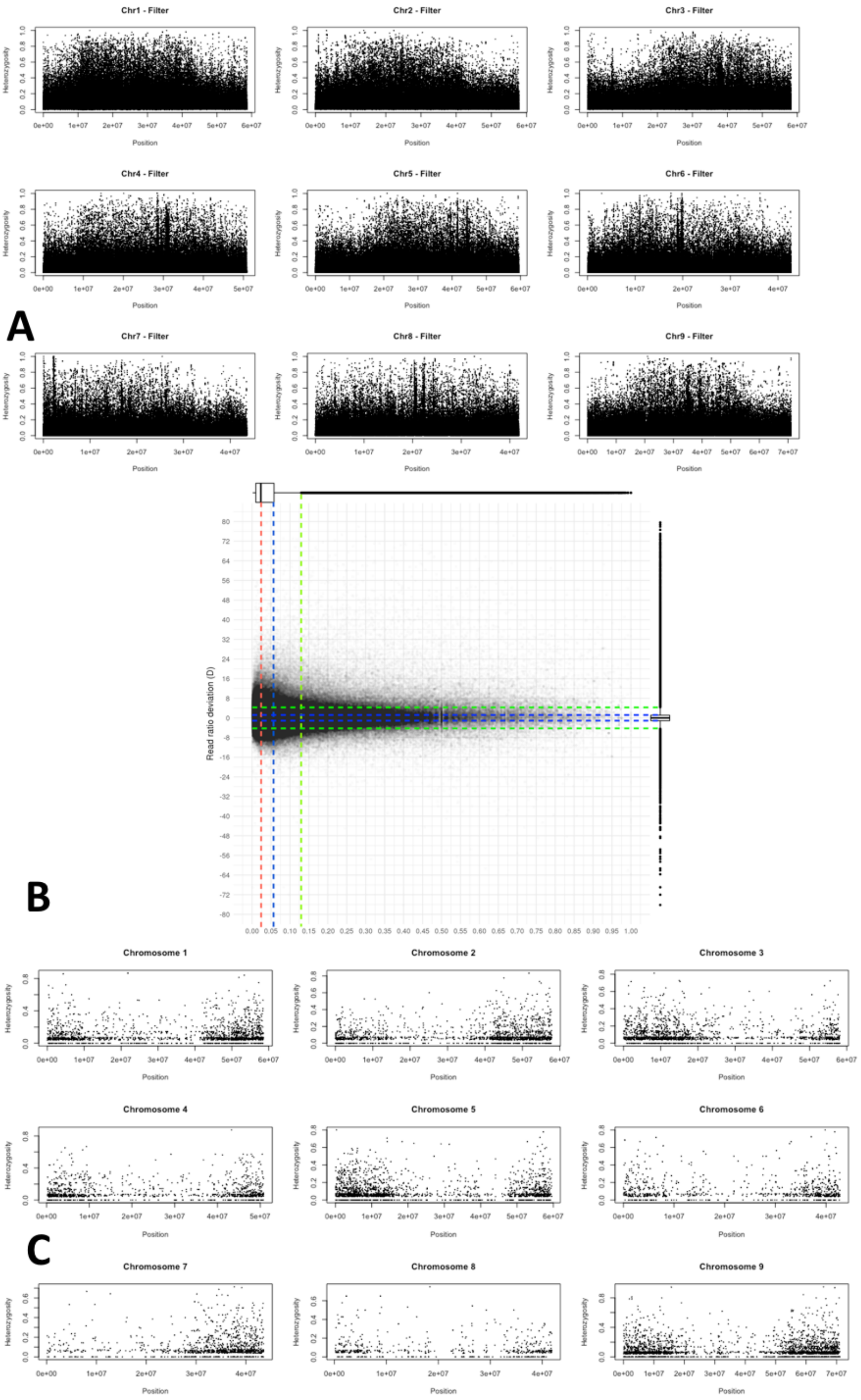

Figure S3. Paralogs filtering *Panicum hallii* var. *hallii* and *Panicum hallii* var. *filipes* data set. A. Heterozygosity by chromosome before filtering by paralogous. B. Number of alleles deviation against the heterozygosity graph. Density of markers represented by boxplot function. C. Heterozygosity after filter by paralogous.

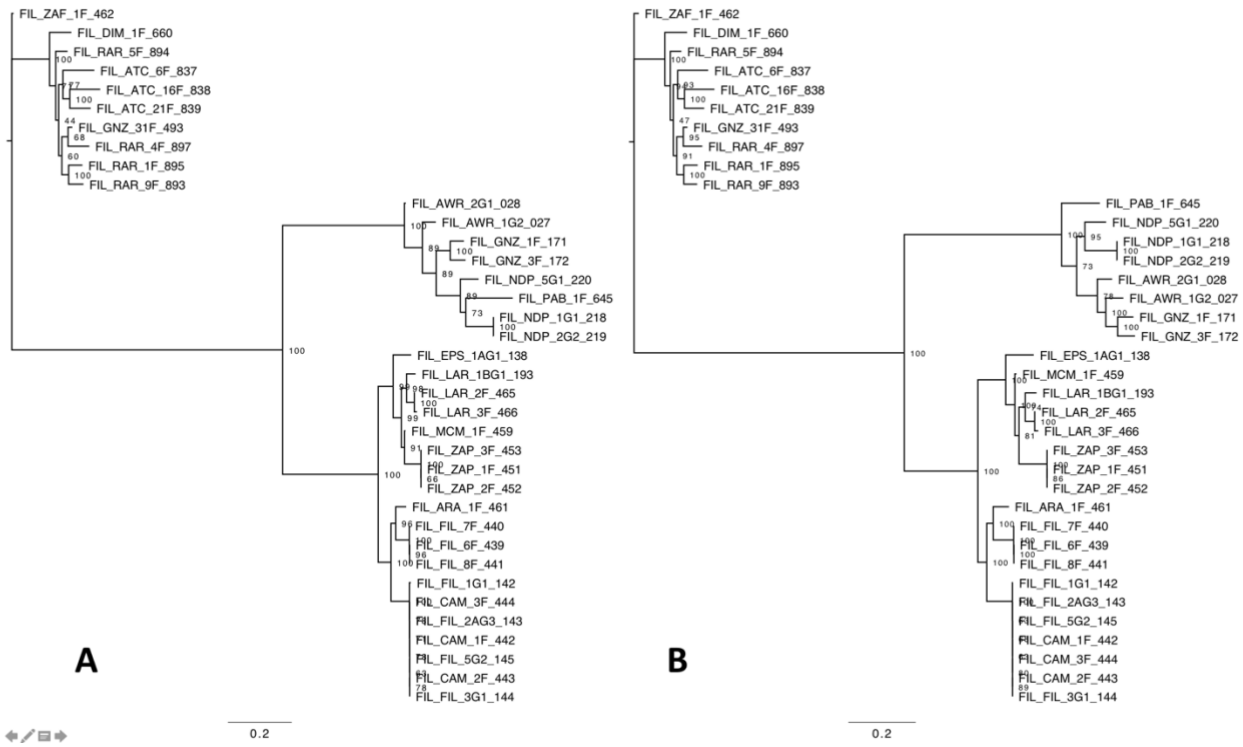

Figure S4. Maximum likelihood phylogenic tree in *Panicum hallii* var. *filipes* using markers mapped against different genome references: A. *Panicum hallii* var. *hallii* v. 2.1 reference genome (92,742 SNPs, 64,814 informative) and, B. *Panicum hallii* var. *filipes* v. 3.1 reference genome (118,027 SNPs, 84,696 informative) (Branch support values after 10,000 boostraps).

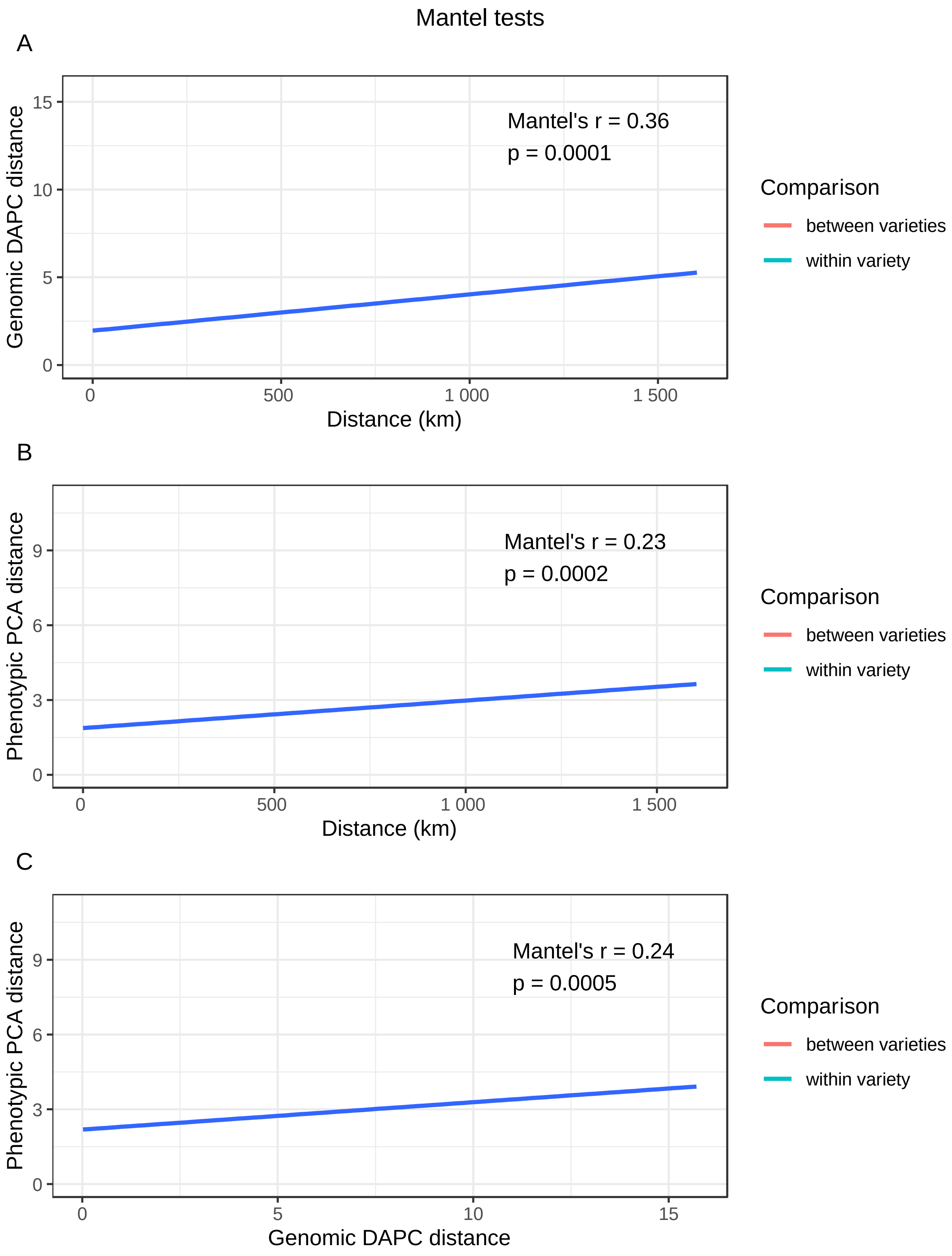
Mantel tests
A
Genomic DAPC distance
15
10
5
0
Mantel's r = 0.36
p = 0.0001
0
500
1 000
1 500
Distance (km)
Comparison
between varieties
within variety
B
Phenotypic PCA distance
9
6
3
0
Mantel's r = 0.23
p = 0.0002
0
500
1 000
1 500
Distance (km)
Comparison
between varieties
within variety
C
Phenotypic PCA distance
9
6
3
0
Mantel's r = 0.24
p = 0.0005
0
5
10
15
Genomic DAPC distance
Comparison
between varieties
within variety

Figure S13: Mantel tests results, A. Between the genetic distances derived from the genomic DAPC and geographic distances, B. Between phenotypic distances derived from the PCA analysis of morphological traits and geographic distances, C. Between phenotypic distances derived from the PCA analysis of morphological traits and the genetic distances derived from the genomic DAPC. Pairwise comparisons between populations of the same variety are shown in sky blue and pairwise comparisons between populations of different varieties are shown in vermillion.

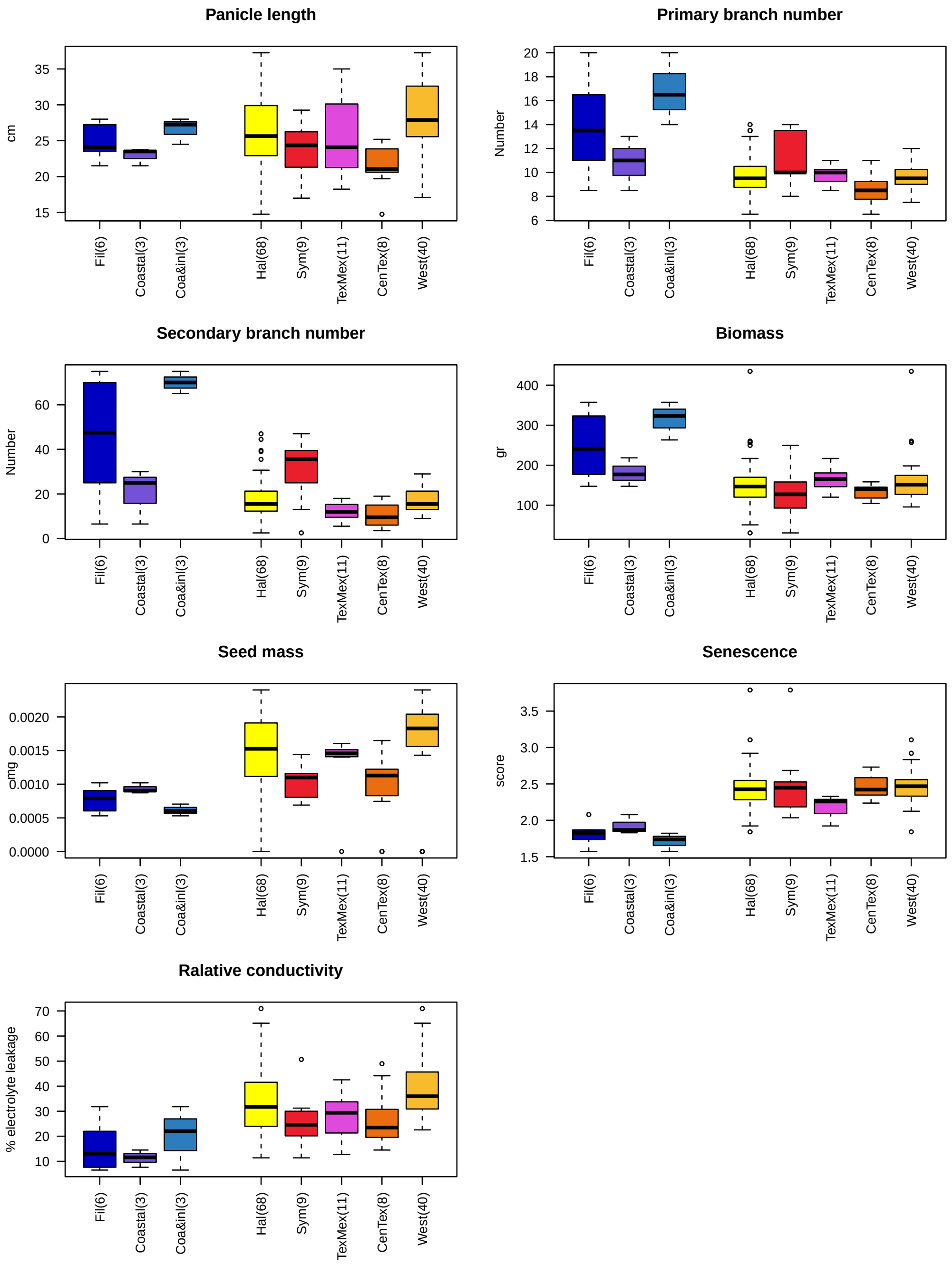

Figure S14 Phenotypic divergence between and within varieties *Panicum hallii* var. *filipes* (Fil) and *Panicum hallii* var. *hallii* (Hal) and between *Panicum hallii* var. *hallii* regions (Coastal, Coas&inl: Coastal and inland, Sym: sympatric, TexMex: Texas Mexico, CenTex: Central Texas, West). Number of individuals in parenthesis.
