# Supplementary material for: Geographic patterns of genomic diversity and structure in the C4 grass Panicum hallii across its natural distribution
Source: AoB Plants. 2021 Jan 6;13(2):plab002. doi: 10.1093/aobpla/plab002 (PMC7937184; doi:10.1093/aobpla/plab002)
Supplement: plab002_suppl_Supplementary_File_2 [file plab002_suppl_supplementary_file_2.pdf]

Geographic patterns of genomic diversity and structure in the C_4 grass *Panicum hallii* across its natural distribution

Supplementary file 2. Habitat Suitability Modeling. Detailed methods and results.

Contents

Methods . . . 1
Species occurrence points . . . 1
Environmental variables . . . 1
Pseudo-absences sampling . . . 2
Habitat Suitability Modelling and ensemble . . . 2
Species distribution hindcast based on habitat suitability models projections . . . 2
Results . . . 3
Panicum hallii var. *filipes* . . . 3
Panicum hallii var. *hallii* . . . 7
References . . . 11

Methods

Species occurrence points

We compiled occurrence localities from direct field surveys, georeferenced herbarium specimens and public databases for biological records. All secondary occurrence points were first verified for probable errors. A total of 965 localities covering the full extant geographic range of *Panicum hallii*, of which 65 come from *P. hallii* var. *filipes* and 904 from *P. hallii* var. *hallii.* We deduplicated the presence data keeping one occurrence per raster cell grid using the function gridSample of the *dismo* R package (Hijmans, Phillips, Leathwick, & Elith, 2017). To reduce potential bias due to uneven sampling effort (Boria, Olson, Goodman, & Anderson, 2014; Kramer-Schadt et al., 2013) we performed a spatial filtering of occurrence points by randomly removing localities that were within 0.4 degrees of one another. We employed the R package *spThin v.0.2.0* (Aiello-Lammens, Boria, Radosavljevic, Vilela, & Anderson, 2015) to retain the maximum number of presences possible under a minimum separation distance. We also included a set of true absence locations that consistently showed no records of *P. hallii* in field surveys between authum 2014 and summer 2017. Using the true absence set points, we created a buffer of 0.4 degrees of the absence points using the function st_buffer of the package *sf* (Pebesma, 2018) and then removed all presence points falling inside the buffer using the function *erase.point* of the package *spatialEco v.1.3-1* (Evans, 2020) in R. The final set of presence points included 36 records for *P. hallii* var. *filipes* and 439 for *P. hallii* var. *hallii.*

Environmental variables

We obtained the explanatory variables for the Habitat Suitability Modeling (HSM) for the present day (1960–1990) from the WorldClim climate archive (Hijmans, Cameron, Parra, Jones, & Jarvis, 2005) at 30 arc-second resolution (ca. 1 km at the Equator). This fine-scale resolution improves predictions for fixed such as plants (Guisan & Thuiller, 2005). In order to avoid multicollinearity-related noise, we performed variable selection using the function corselect of the R package *fuzzySim* (Barbosa, 2015) by calculating pairwise correlations among the variables and, among each pair of variables with Pearson correlation above 0.75, we keep the variable with the highest bivariate (individual) relationship with species presence/absence response in a binomial Generalized Linear model. All final explanatory data sets include eight bioclimatic variables for each fitted model, in all cases the variation inflation factors were below 10. We cropped the bioclimatic layers from latitude 13° to 44 °N and longitude 88° to 120°W with *raster* R package (Hijmans, 2015), this geographic extent include the full extant range of *P. hallii.*

Pseudo-absences sampling

Because the HSM techniques that we implemented required both presence and absence data, we selected pseudo-absences as random localities throughout the full extant range of the species. For each independent modeling run for each variety, we set five samples of 10000 random localities outside a buffer of 0.4 degrees of the full set of presence/absence points. We generated the spatial buffer with st_buffer function and the transformed to a raster layer using the *fasterize* package in R (Ross, 2020).

- **Occurence sampling**

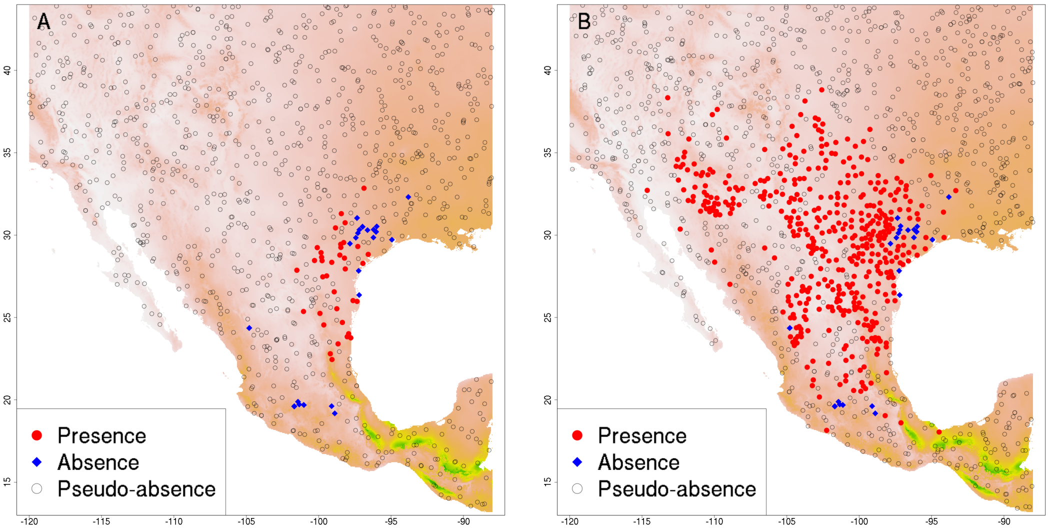

Figure S2. Sampling points for presence, absence, and pseudo-absences locations for **A.** *P. hallii* var. *filipes* and **B.** *P. hallii* var. *hallii*. The plot shows a subset of 1000 out of 50000 pseudo-absence points sampled for the modeling processes. Background colors (green to white) represent Annual Precipitation values from 35 to 5375 mm.

Habitat Suitability Modelling and ensemble

We implemented an ensemble-HSM framework (Araujo & New, 2007) that use models obtained by several algorithms to account for the uncertainty associated with the HSM techniques. The analyses were implemented with *biomod2* package (Thuiller, Georges, & Engler, 2014; Thuiller, Lafourcade, Engler, & Araújo, 2009). Individual HSMs were generated using seven modelling techniques: (1) Generalized Linear Models (GLM; McCullagh & Nelder, 1983), (2) Generalized Additive Models (GAM; Hastie & Tibshirani, 1990), (3) Generalized Boosting Models (GBM; Death, 2007), (4) Classification Tree Analysis (CTA; Breiman, Friedman, Stone, & Olshen, 1993), (5) Multiple Adaptive Regression Splines (MARS; Leathwick, Rowe, Richardson, Elith, & Hastie, 2005), (6) Random Forests (RF; Breiman, 2001), and (7) Maximum Entropy (MaxEnt; Phillips, Anderson, & Schapire, 2006). We generated independent HSM for *P. hallii* var. *hallii*, *P. hallii* var. *hallii* – West genetic cluster, *P. hallii* var. *hallii* – Tex-Mex genetic cluster and *P. hallii* var. *filipes*. We kept prevalence equal to 0.5. For each HSM, we run five cross-validation replicated where presence localities were divided in sets of 75% for training models and 25% for testing. For each HSM we performed five runs for each of the pseudo-absence sets, therefore we completed a total of 175 HSM for each *P. hallii* variety/cluster. To assess predictive performance of the HSM, we measured the threshold independent statistics area under the receiver operating characteristic curve (AUC; Phillips et al., 2006) and the True Skill Statistic (TSS; Allouche, Tsoar, & Kadmon, 2006). From the full set of obtained HSMs for each *P. hallii* variety/cluster we calculated an ensemble-HSM (i.e. consensus model) using a mean of suitability scores weighted by the TSS evaluation score of each individual HSM. To calculate de consensus, we only included HSMs with TSS > 0.75.

Species distribution hindcast based on habitat suitability models projections

We projected the fitted HSM to infer the distribution of P. hallii during three past conditions: Mid-Holocene (midHol; ~6 Kya), Last Glacial Maximum (LGM; ~22 Kya) and Last Inter-Glacial (LIG; ~120-140 Kya) periods. To take into account the uncertainty related to different approaches to simulate past climate scenarios, for midHol and LGM period we projected the HSMs to three different global circulation models (GCMs): MIROC (Model for Interdisciplinary Research on Climate; Hasumi & Emori, 2004), CCSM4 (The Community Climate System Model

Version 4; Gent et al., 2011), and MPI-ESM-P (Max–Planck-Institute Earth System Model; Giorgetta et al., 2013). For past climate projections, we approximated a consensus distribution map ranges to the areas that were predicted by one, two, or three binary-transformed ensemble HSMs for both different circulation model hindcasts. Map for HSM projection visualization were produced with the packages rasterVis and viridis packages in R (Garnier, 2018; Perpiñán & Hijmans, 2019).

Results

Panicum hallii var. *filipes*

- **Individual model evaluations**

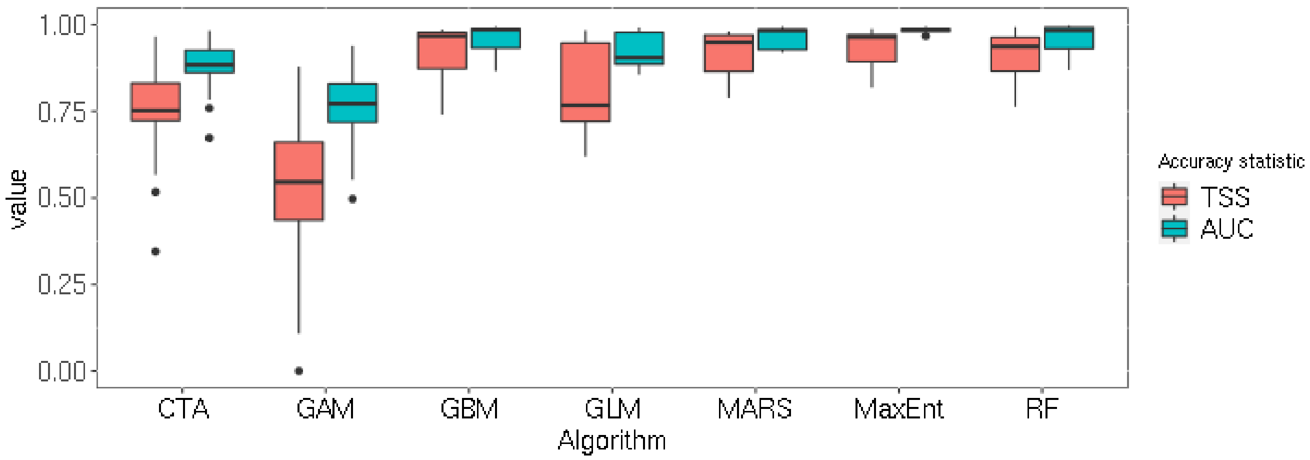

Figure S5. Boxplots for the individual model evaluation statistics Area under the receiver operating characteristic curve (AUC) and the True Skill Statistic (TSS) for the habitat suitability modeling for *P. hallii* var. *filipes.*"

* **Bioclimatic variable selection**

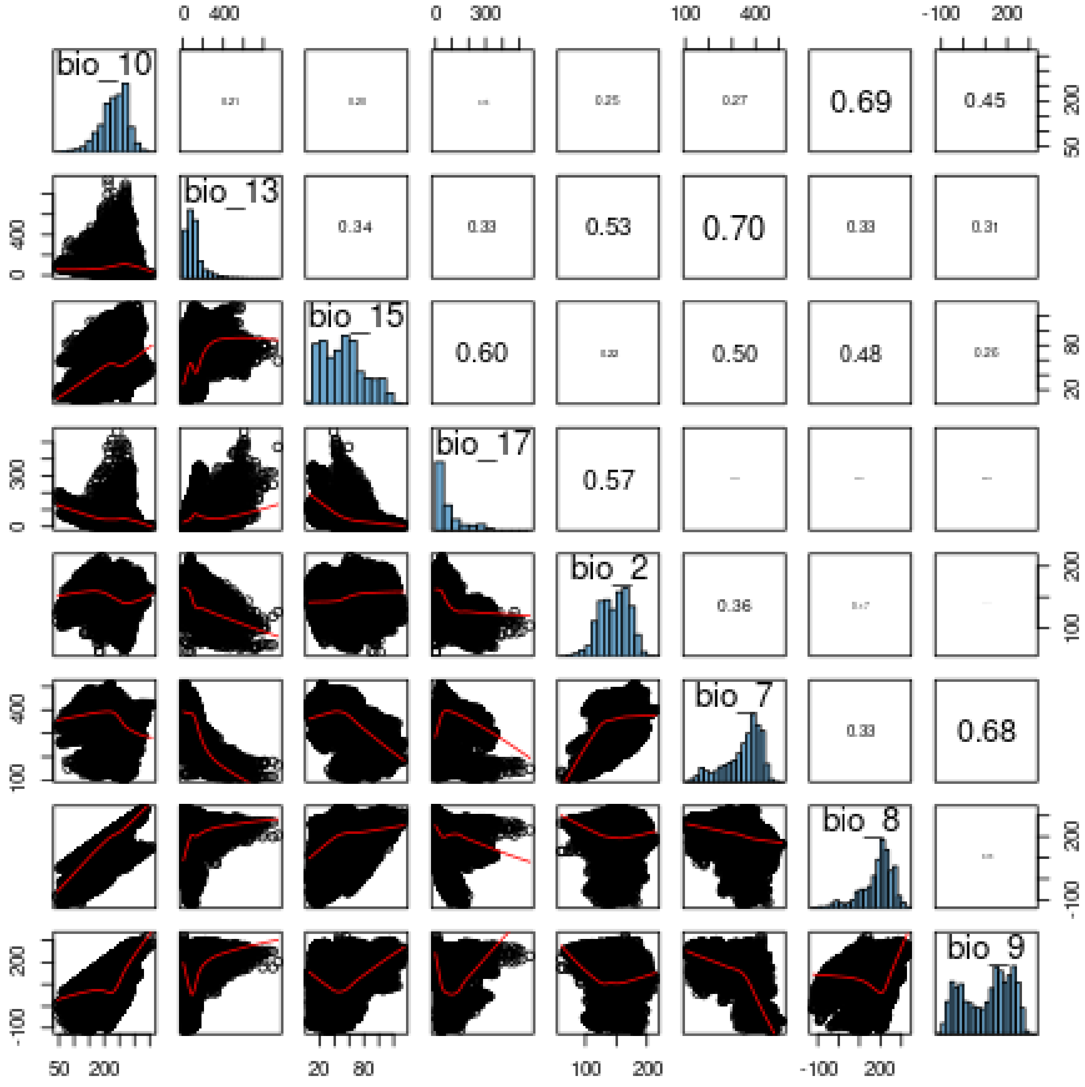

Figure S6. Correlation matrix of bioclimatic selected variables for the habitat suitability modeling for *P. hallii* var. *filipes*."

* **Individual variable importance**

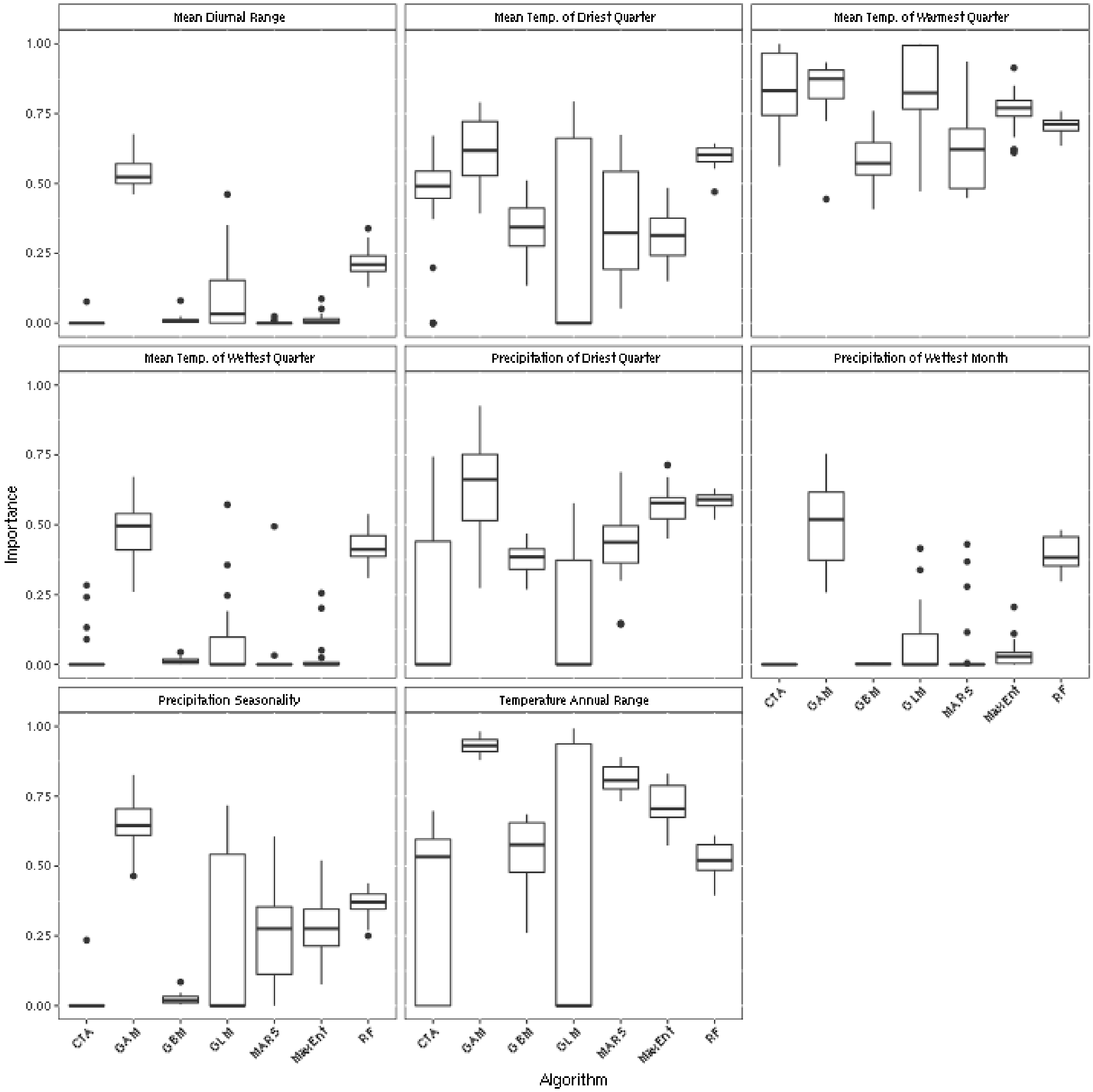

Figure S7. Variable importance for the habitat suitability modeling for *P. hallii* var. *filipes*.

* **Ensemble habitat suitability model projections**

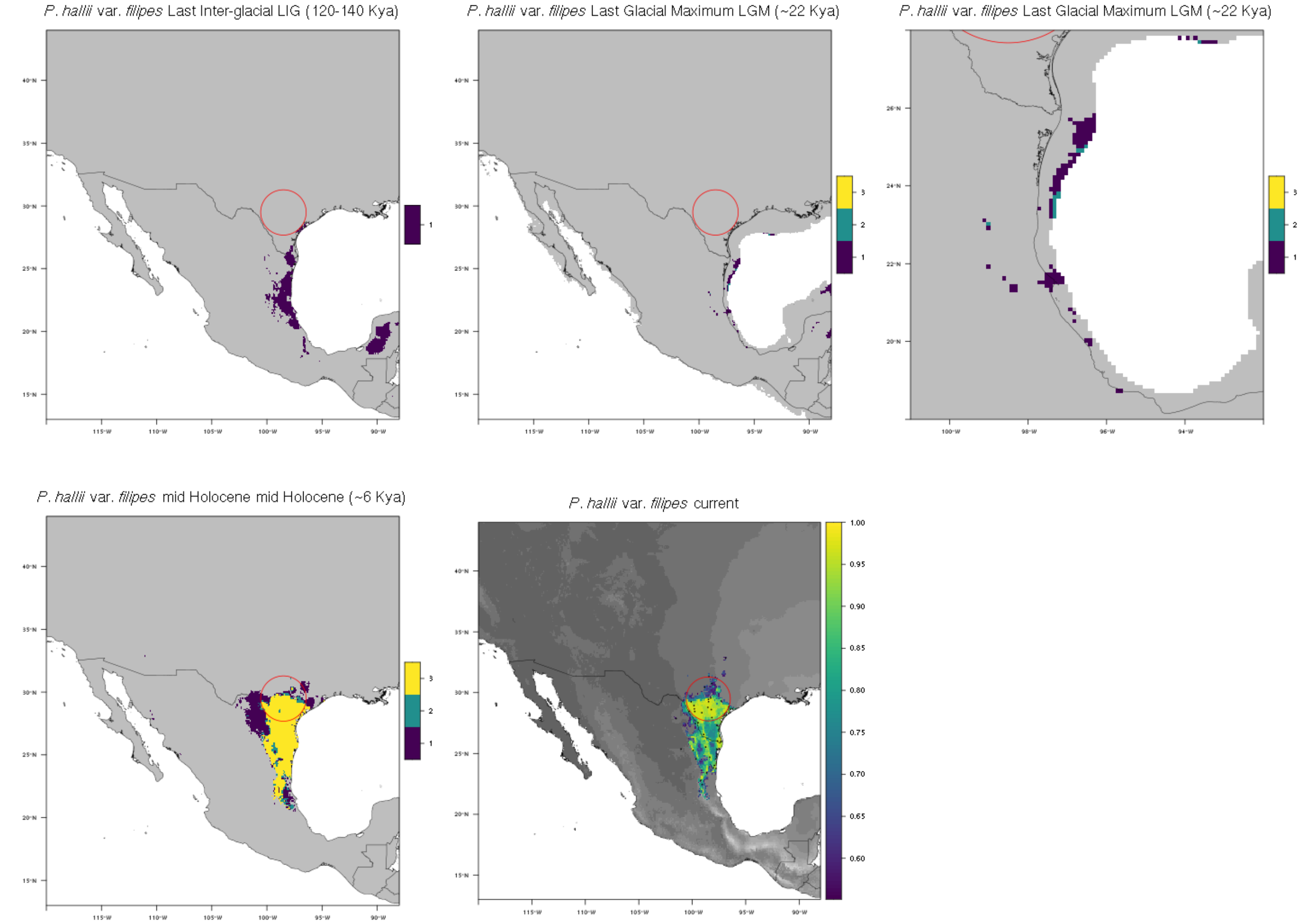

Figure S8. Projections of the ensemble habitat suitability model of *P. hallii* var. *filipes* to Last Inter-Glacial, Last Glacial Maximum, mid-Holocene and Current climatic conditions. Color categorical scale in the paleo-climatic projections represents the climatic suitable area predicted by either one, two or three GCMs for the specific period above the TSS threshold (see Methods). Color continuous scale in the current projection represents the suitability values projected from the HSM above the TSS threshold. Background gray scale in the current projection represents the Annual Precipitation gradient of the studied area from 35 mm (dark) to 5375 mm (ligth). The red circle represents the *P. hallii* diversity hotspot as a radius of 200 km around San Antonio, Texas.

Panicum hallii* var. *hallii

- **Individual model evaluations**

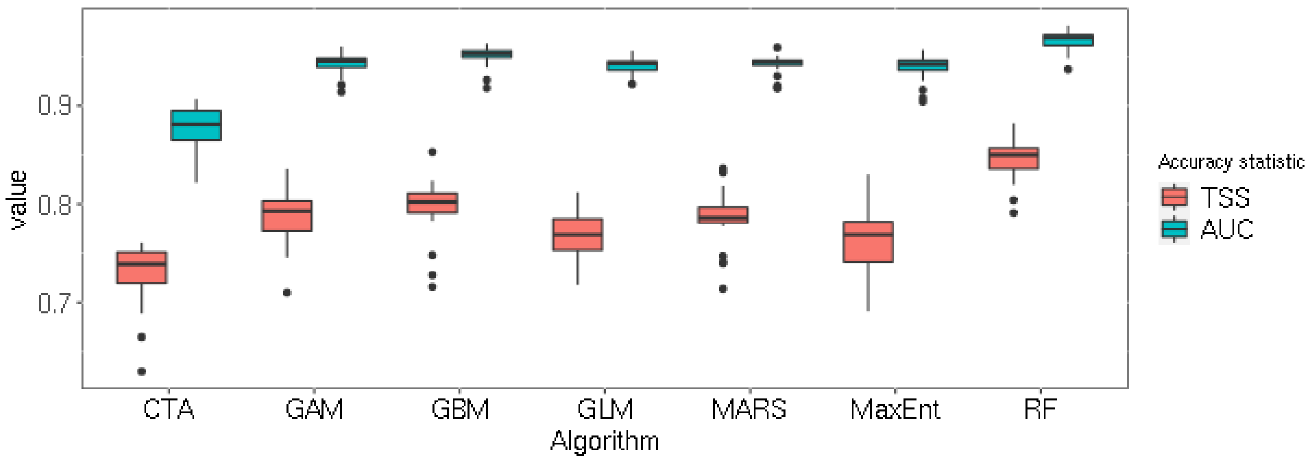

"**Figure S9.** Boxplots for the individual model evaluation statistics Area under the receiver operating characteristic curve (AUC) and the True Skill Statistic (TSS) for the habitat suitability modeling for *P. hallii* var. *hallii*.

- **Bioclimatic variable selection**

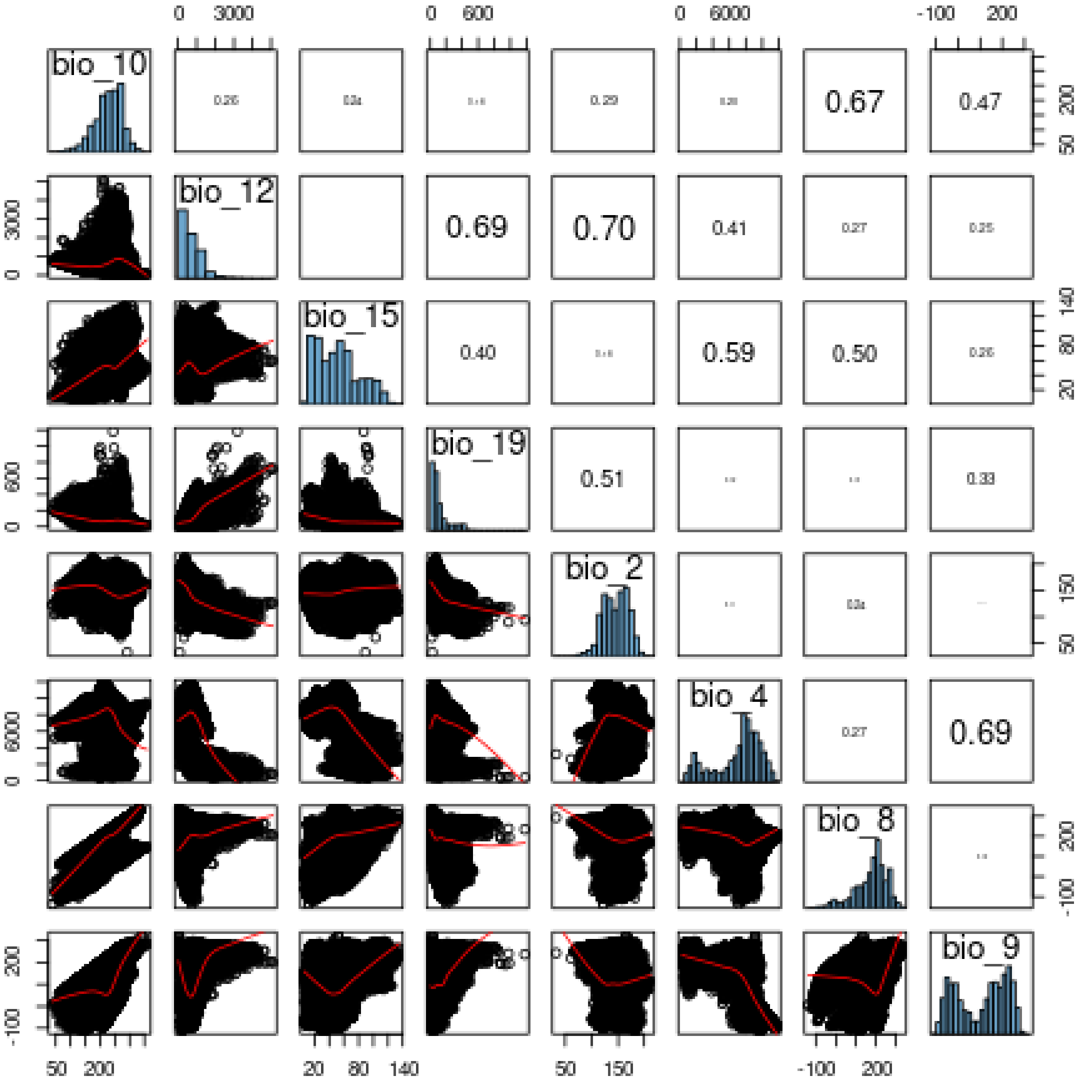

Figure S10. Correlation matrix of bioclimatic selected variables for the habitat suitability modeling for *P. hallii* var. *hallii*.

- **Individual variable importance**

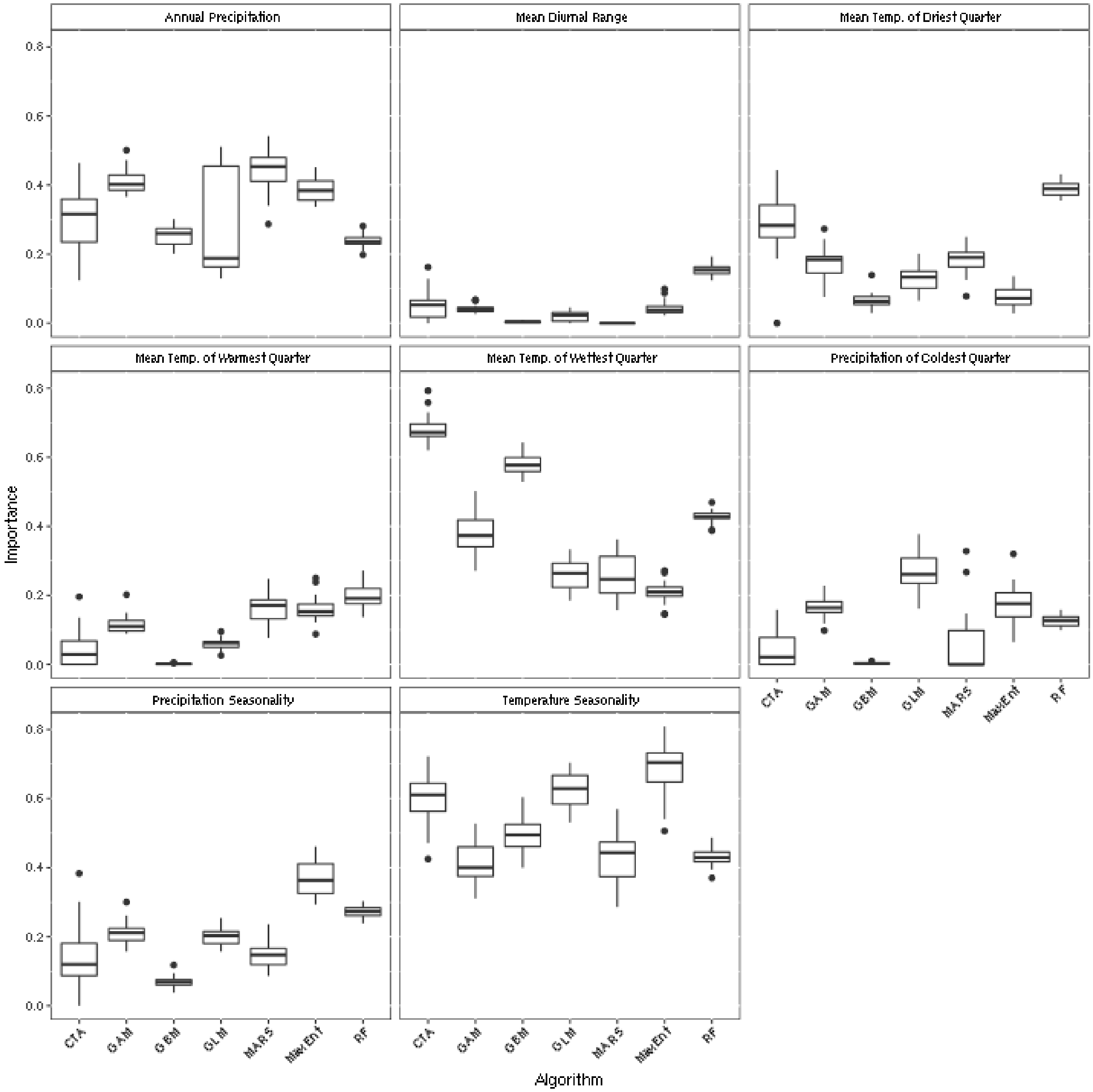

Figure S11. Variable importance for the habitat suitability modeling for *P. hallii* var. *hallii*.

- **Ensemble habitat suitability model projections**

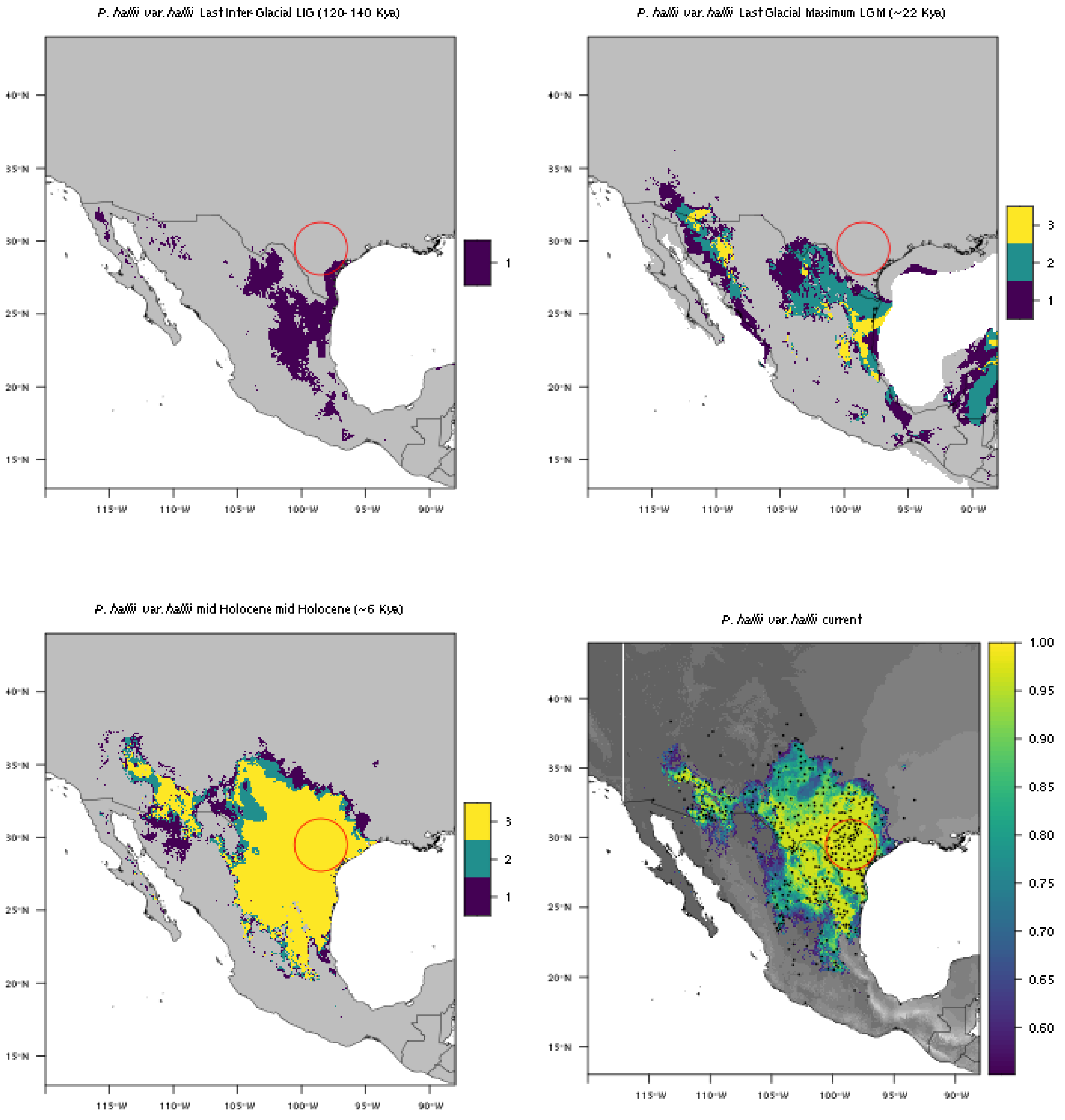

Figure S12. Projections of the ensemble habitat suitability model of *P. hallii* var. *hallii* to Last Inter-Glacial, Last Glacial Maximum, mid-Holocene and Current climatic conditions. Color categorical scale in the paleo-climatic projections represents the climatic suitable area predicted by either one, two or three GCMs for the specific period above the TSS threshold (see Methods). Color continuous scale in the current projection represents the suitability values projected from the HSM above the TSS threshold. Background gray scale in the current projection represents the Annual Precipitation gradient of the studied area from 35 mm (dark) to 5375 mm (ligth). The red circle represents the *P. hallii* diversity hotspot as a radius of 200 km around San Antonio, Texas.

References

Aiello-Lammens, M. E., Boria, R. A., Radosavljevic, A., Vilela, B., & Anderson, R. P. (2015). spThin: An R package for spatial thinning of species occurrence records for use in ecological niche models. *Ecography*, *38*(5), 541–545. doi: 10.1111/ecog.01132

Allouche, O., Tsoar, A., & Kadmon, R. (2006). Assessing the accuracy of species distribution models: Prevalence, kappa and the true skill statistic (TSS). *Journal of Applied Ecology*, *43*(6), 1223–1232. doi: 10.1111/j.1365-2664.2006.01214.x

Araujo, M., & New, M. (2007). Ensemble forecasting of species distributions. *Trends in Ecology & Evolution*, *22*(1), 42–47. doi: 10.1016/j.tree.2006.09.010

Barbosa, A. M. (2015). fuzzySim: Applying fuzzy logic to binary similarity indices in ecology. *Methods in Ecology and Evolution*, *6*(7), 853–858. doi: 10.1111/2041-210X.12372

Boria, R. A., Olson, L. E., Goodman, S. M., & Anderson, R. P. (2014). Spatial filtering to reduce sampling bias can improve the performance of ecological niche models. *Ecological Modelling*, *275*, 73–77. doi: 10.1016/j.ecolmodel.2013.12.012

Breiman, L. (2001). Random forests. *Machine Learning*, *45*(1), 5–32. doi: 10.1023/A:1010933404324

Breiman, L., Friedman, J., Stone, C. J., & Olshen, R. A. (1993). *Classification and regression trees.* New York, N.Y.: Chapman & Hall.

Death, G. (2007). Boosted trees for ecological modeling and prediction. *Ecology*, *88*(1), 243–251. doi: 10.1890/0012-9658(2007)88[243:BTFEMA]2.0.CO;2

Evans, J. S. (2020). *spatialEco.* Retrieved from https://github.com/jeffreyevans/spatialEco

Garnier, S. (2018). *Viridis: Default color maps from 'matplotlib'.* Retrieved from https://CRAN.R-project.org/package=viridis

Gent, P. R., Danabasoglu, G., Donner, L. J., Holland, M. M., Hunke, E. C., Jayne, S. R., … Zhang, M. (2011). The Community Climate System Model Version 4. *Journal of Climate*, *24*(19), 4973–4991. doi: 10.1175/2011JCLI4083.1

Giorgetta, M. A., Jungclaus, J., Reick, C. H., Legutke, S., Bader, J., Böttinger, M., … Stevens, B. (2013). Climate and carbon cycle changes from 1850 to 2100 in MPI-ESM simulations for the Coupled Model Intercomparison Project phase 5: Climate Changes in MPI-ESM. *Journal of Advances in Modeling Earth Systems*, *5*(3), 572–597. doi: 10.1002/jame.20038

Guisan, A., & Thuiller, W. (2005). Predicting species distribution: Offering more than simple habitat models. *Ecology Letters*, *8*(9), 993–1009. doi: 10.1111/j.1461-0248.2005.00792.x

Hastie, T. J., & Tibshirani, R. J. (1990). *Generalized additive models.* London, England: Chapman & Hall/CRC.

Hasumi, H., & Emori, S. (2004). *K-1 coupled GCM (MIROC) description.* University of Tokyo, Tokyo: Center for Climate System Research. Retrieved from http://www.ccsr.u-tokyo.ac.jp/kyosei/hasumi/MIROC/techrepo.pdf

Hijmans, R. J. (2015). *Raster: Geographic data analysis and modeling. R package version 2.3-40. Http://CRAN.R-project.org/package=raster.* Retrieved from http://CRAN.R-project.org/package=raster

Hijmans, R. J., Cameron, S. E., Parra, J. L., Jones, P. G., & Jarvis, A. (2005). Very high resolution interpolated climate surfaces for global land areas. *International Journal of Climatology*, *25*(15), 1965–1978. doi: 10.1002/joc.1276

Hijmans, R. J., Phillips, S. J., Leathwick, J., & Elith, J. (2017). *Dismo: Species Distribution Modeling.* Retrieved from https://CRAN.R-project.org/package=dismo

Kramer-Schadt, S., Niedballa, J., Pilgrim, J. D., Schröder, B., Lindenborn, J., Reinfelder, V., … Wilting, A. (2013). The importance of correcting for sampling bias in MaxEnt species distribution models. *Diversity and Distributions*, *19*(11), 1366–1379. doi: 10.1111/ddi.12096

Leathwick, J. R., Rowe, D., Richardson, J., Elith, J., & Hastie, T. (2005). Using multivariate adaptive regression splines to predict the distributions of New Zealand's freshwater diadromous fish. *Freshwater Biology*, *50*(12), 2034–2052. doi: 10.1111/j.1365-2427.2005.01448.x

McCullagh, P., & Nelder, J. A. (1983). *Generalized linear models.* London, England: Chapman; Hall/CRC.

Pebesma, E. (2018). Simple features for R: Standardized support for spatial vector data. *The R Journal*, *10*(1), 439. doi: 10.32614/RJ-2018-009

Perpiñán, O., & Hijmans, R. (2019). *rasterVis.* Retrieved from http://oscarperpinan.github.io/rastervis/

Phillips, S. J., Anderson, R. P., & Schapire, R. E. (2006). Maximum entropy modeling of species geographic distributions. *Ecological Modelling*, *190*(3-4), 231–259. doi: 10.1016/j.ecolmodel.2005.03.026

Ross, N. (2020). *Fasterize: Fast Polygon to Raster Conversion.* Retrieved from https://CRAN.R-project.org/package=fasterize

Thuiller, W., Georges, D., & Engler, R. (2014). *Biomod2: Ensemble platform for species distribution modeling.* Retrieved from http://R-Forge.R-project.org/projects/biomod/

Thuiller, W., Lafourcade, B., Engler, R., & Araújo, M. B. (2009). Biomod - a platform for ensemble forecasting of species distributions. *Ecography*, *32*(3), 369–373. doi: 10.1111/j.1600-0587.2008.05742.x
